# Cigarette smoking is associated with an altered vaginal tract metabolomic profile

**DOI:** 10.1038/s41598-017-14943-3

**Published:** 2018-01-16

**Authors:** T. M. Nelson, J. C. Borgogna, R. D. Michalek, D. W. Roberts, J. M. Rath, E. D. Glover, J. Ravel, M. D. Shardell, C. J. Yeoman, R. M. Brotman

**Affiliations:** 1 Department of Animal and Range Sciences, Montana State University, Bozeman, MT USA; 2 Department of Microbiology and Immunology, Montana State University, Bozeman, MT USA; 3 Metabolon Inc, Durham, NC USA; 4 Department of Ecology, Montana State University, Bozeman, MT USA; 5 Department of Behavioral and Community Health, University of Maryland School of Public Health, College Park, MD USA; 6 Truth Initiative, Washington DC, USA; 7 Institute for Genome Sciences, University of Maryland School of Medicine, Baltimore, MD USA; 8 Department of Microbiology and Immunology, University of Maryland School of Medicine, Baltimore, MD USA; 9 Translational Gerontology Branch, National Institute on Aging, Baltimore, MD USA; 10 Department of Epidemiology and Public Health, University of Maryland School of Medicine, Baltimore, MD USA

## Abstract

Cigarette smoking has been associated with both the diagnosis of bacterial vaginosis (BV) and a vaginal microbiota lacking protective *Lactobacillus* spp. As the mechanism linking smoking with vaginal microbiota and BV is unclear, we sought to compare the vaginal metabolomes of smokers and non-smokers (17 smokers/19 non-smokers). Metabolomic profiles were determined by gas and liquid chromatography mass spectrometry in a cross-sectional study. Analysis of the 16S rRNA gene populations revealed samples clustered into three community state types (CSTs) ---- CST-I (*L*. *crispatus*-dominated), CST-III (*L*. *iners*-dominated) or CST-IV (low-*Lactobacillus*). We identified 607 metabolites, including 12 that differed significantly (q-value < 0.05) between smokers and non-smokers. Nicotine, and the breakdown metabolites cotinine and hydroxycotinine were substantially higher in smokers, as expected. Among women categorized to CST-IV, biogenic amines, including agmatine, cadaverine, putrescine, tryptamine and tyramine were substantially higher in smokers, while dipeptides were lower in smokers. These biogenic amines are known to affect the virulence of infective pathogens and contribute to vaginal malodor. Our data suggest that cigarette smoking is associated with differences in important vaginal metabolites, and women who smoke, and particularly women who are also depauperate for *Lactobacillus* spp., may have increased susceptibilities to urogenital infections and increased malodor.

## Introduction

According to the U.S. National Health Interview Survey, 13.9 percent of U.S. women reported that they smoked cigarettes in 2016^1^, and it has been well-documented that women who smoke have greater risks for adverse reproductive health outcomes, including premature birth, delivery of low birth weight infants, certain birth defects, and ectopic pregnancy^2–6^. Smokers are also more susceptible to bacterial infections than non-smokers^7^, however, few studies have investigated the mechanisms linking smoking and gynecologic infections. Smoking has been shown in large observational studies to be a dose-dependent risk factor for the diagnosis of bacterial vaginosis (BV)^8–13^, as well as significantly associated with risk of other genital infections in females including *Trichomonas vaginalis*^14^, herpes simplex virus type 2 (HSV-2)^15^ and *Chlamydia trachomatis*^16,17^. Smoking has long been implicated in higher risk for oral and genital human papillomavirus (HPV) prevalence and viral load, progression to cervical pre-cancer, and vulval intraepithelial neoplasia^18–23^. Smoking is associated with damaged cervical epithelium through DNA modification and suppressed local and systemic immune responses^24^, both of which may increase susceptibility to a wide range of female reproductive tract infections^25,26^. Nicotine’s major metabolite, cotinine, has been shown to become concentrated in cervical mucus, providing evidence that smoking can directly affect the vaginal and cervical epithelium^27,28^. Smoking cessation was also associated with dramatic changes in the gut microbiota in one study^29^.

In a 2014 study, our research group confirmed that the composition of the vaginal microbiota is strongly associated with smoking^30^. In that study, we compared the vaginal microbiota, as determined by 16S rRNA gene amplicon sequencing, between smokers and non-smokers. We reported that women who had a vaginal microbiota that were lacking significant numbers of *Lactobacillus* spp. were 25-fold more likely to report current smoking than women with a *L*. *crispatus*-dominated microbiota. Most *Lactobacillus* spp. are thought to provide broad-spectrum protection to pathogenic infections through the production of lactic acid and bacteriocins^31,32^. The lactic acid reduces the vaginal pH to ≤4.0 and is a potent bactericide and virucide^31,33–36^. Indeed, epidemiologic studies have shown that a relatively low level of *Lactobacillus* spp. and the presence of a wide array of strict and facultative anaerobes in the vaginal microbiota, as observed in the clinical diagnosis of BV, is associated with increased incidence of HIV and other sexually transmitted infections (STIs)^15,37–43^.

Despite advances in understanding the epidemiologic links between smoking and women’s reproductive health, the mechanistic processes by which smoking affects the vaginal microenvironment and BV is unclear. The effect of smoking on the function of the microbiome can be investigated by assessing the metabolome. The metabolome is the set of small (<1 kDa) molecule chemicals, which includes host and microbially-produced and modified biomolecules, as well as exogenous chemicals^44^. Measurement of the metabolomic profile from any biological sample is expected to contain numerous low molecular mass molecules with different physicochemical characteristics and concentration ranges^44^.

The metabolome is an important characteristic of the vaginal microenvironment and differences in some metabolites are associated with functional variations of the vaginal microbiota^45–48^. For example, the presence of certain chemicals or metabolites have been shown *in vitro* to directly reduce or inhibit the growth of select bacterial species^49–51^. Biosynthesis of biogenic amines (BAs) (cadaverine, putrescine, spermine, spermidine, trimethylamine, and tyramine) may allow various bacteria to survive in low pH environments^52^, like that found in the healthy *Lactobacillus*-dominated vagina. BAs have also been shown to enhance growth rates of various pathogenic bacteria, such as *Neisseria gonorrhoeae*, and shield them from host innate immunological defenses^53–55^. The recent applications of metabolomics with *in vivo* samples have identified relationships between bacterial species and vaginal metabolomic profiles. In particular *Dialister*, *Mobiluncus*, *Atopobium*, *Prevotella*, *Mycoplasma* and *Gardnerella* species were correlated with the presence of several metabolites along with vaginal odor and discharge^45–48^. We have hypothesized that BAs may be an important feature in the destabilization of *Lactobacillus* spp.-dominant vaginal microbiota and the initiation of BV, as well as the characteristic malodor of BV^56^. The decarboxylation of amino acid precursors to form BAs results in increased pH and may increase the risk for BV^57^.

In this study, we sought to characterize vaginal microbiota functional differences between smokers and non-smokers by investigating the vaginal metabolome. These data may allow enhanced understanding of the mechanism by which smoking may increase the risk of urogenital infections and may contribute to our understanding of the effect of smoking on the female reproductive tract.

## Results

**Figure 1:**
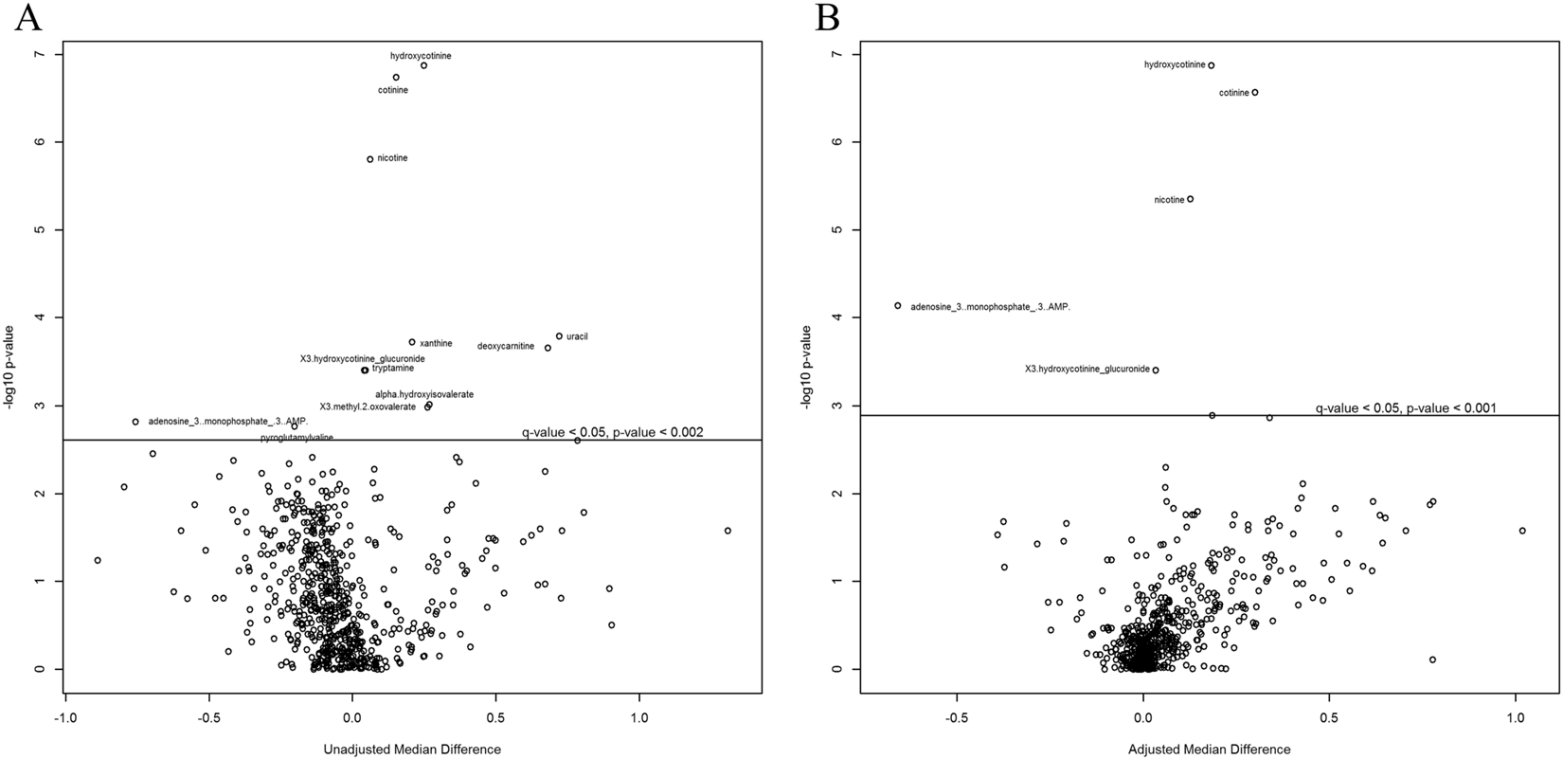
Compounds differing between smokers and non-smokers with and without adjustment for CST. Volcano plots display –log_10_ (p-value) and the median difference in concentration between smokers and non-smokers when unadjusted (**A**) and adjusted for community state type (**B**). Quantile regression was conducted on centered and scaled metabolite concentrations. Significance testing was conducted with Wilcoxon rank sum test and corrected for multiple comparisons. Metabolites that differed significantly where q-value < = 0.05 between smokers and non-smokers are shown above the line in each plot.

**Figure 2:**
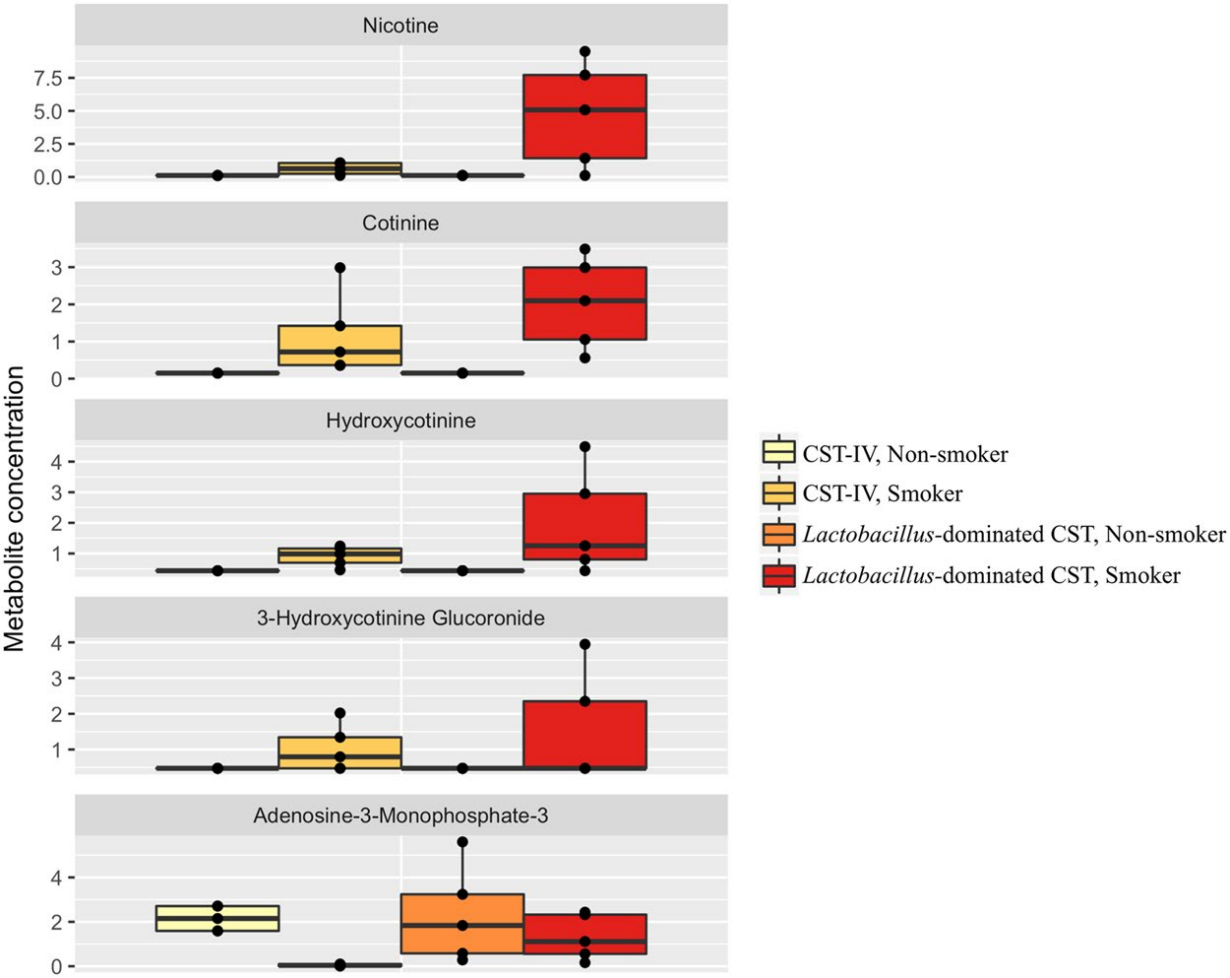
Vaginal metabolites that differ between smokers and non-smokers. Boxplots display metabolites identified as significantly (q-value = <0.05) different in the vagina of smokers and non-smokers when unadjusted and adjusted for the impact of bacterial community state type (CST). Samples categorized as CST-I (*L*. *crispatus*-dominated) and CST-III (*L*. *iners*-dominated) were grouped and compared with CST-IV (low-*Lactobacillus* spp.). Quantile regression was conducted on centered and scaled metabolite concentrations. Significance testing was conducted with Wilcoxon rank sum test and corrected for multiple comparisons.

In total, 607 compounds were identified in the vaginal metabolome of 36 women. PCA indicated that the metabolomic profiles clustered by both smoking status and bacterial community state type (CST) (Figure S1). CST accounted for 23% of the metabolite variation observed in the complete data set (distance based linear model, DISTLM F = 9.9397, P_PERM_ = 0.0001; Table 1), while smoking status explained 6%. However, the concentrations of 12 metabolites were significantly different between smokers and non-smokers (q-value = <0.05; Fig. 1A, Table S1). Tobacco constituents and their primary breakdown products (nicotine, cotinine and hydroxycotinine) represented the strongest differences between smokers and non-smokers with non-smokers having a 2- to 12-fold reduction in these compounds (q-value = <0.05). After adjusting for the influence of CST, five of the metabolites persisted in their differences between smokers and non-smokers (Figs 1B, 2).

**Table 1:** Significantly fitted predicator variables to vaginal metabolites.

Variable	SS (trace)	Pseudo-F	p-value	Proportion (%)	Cumulative Proportion (%)
**Individual model test**					
CST	3181.00	9.94	0.002	22.62	NA
Smoking status	880.65	2.27	0.074	6.26	NA
Race	1120.00	2.94	0.033	7.96	NA
Education level	1216.90	3.22	0.038	8.65	NA
**Fitted model with selected variables**, **adjusted R**^**2**^** = 0**.**26**					
CST	3181.00	9.94	0.001	22.62	22.62
Race	817.71	2.68	0.037	5.82	28.44
Education level	533.18	1.79	0.11	3.79	32.23
Smoking status	292.20	0.98	0.37	2.08	34.31

**Figure 3:**
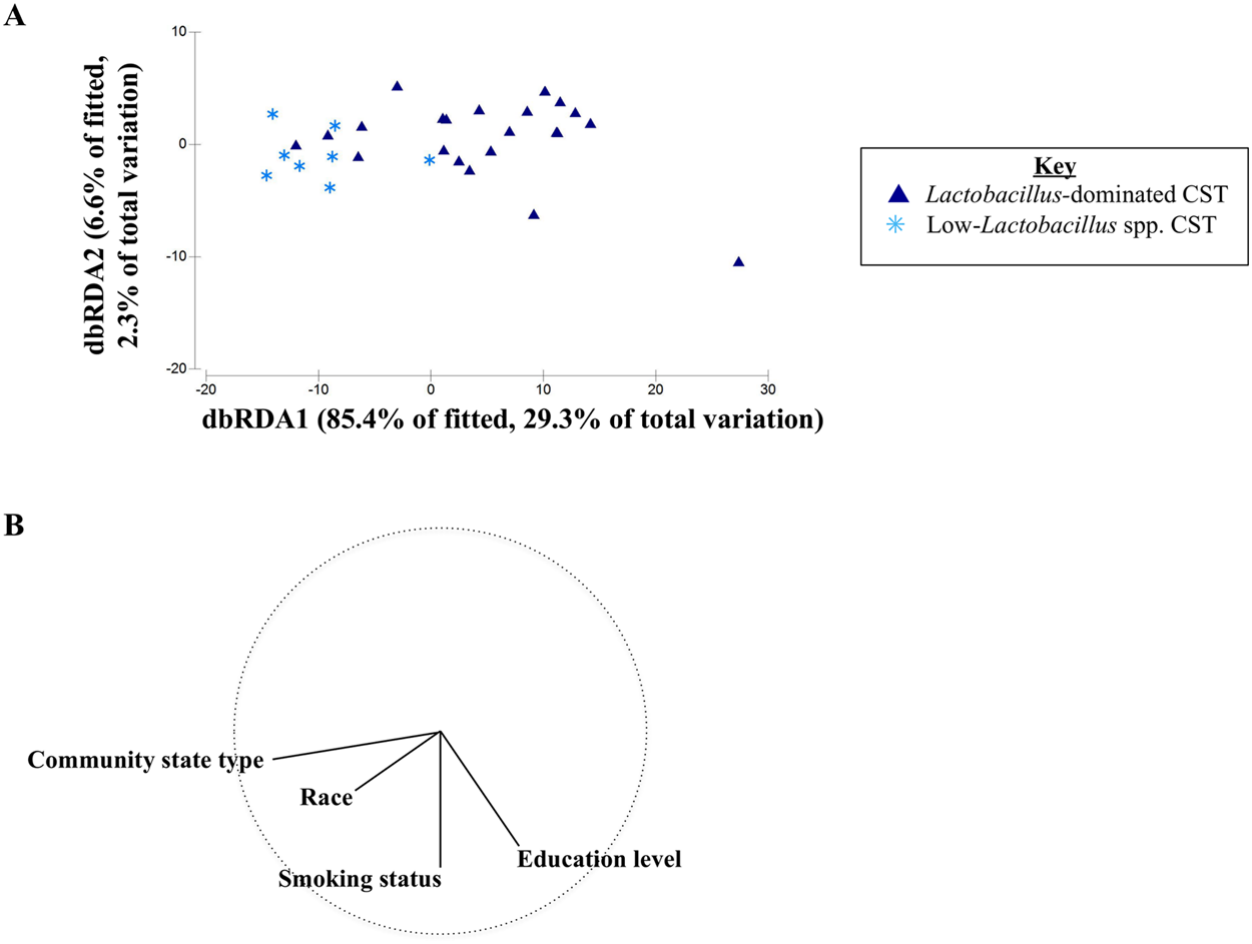
Participant predictor variables fitted to vaginal metabolites. Distance-based linear modelling (DISTLM) was conducted on log-transformed Euclidean distance matrix of vaginal metabolites. All variables were included in the final model that when applied to the data cloud of vaginal metabolites (Table 1). Distance-based redundancy analysis (dbRDA) plot (**A**) displays the metabolite composition fitted to the four variables labeled with the strongest variable, CST. Variable vectors indicating strength and direction are identified (**B**).

Distance based linear modelling (DISTLM) was performed on vaginal metabolites fitted with participant behavioral variables listed in Table 2. DISTLM was conducted with adjusted R2 over 9,999 permutations. DISTLM identifies the best-fit model based on all the available variables that best-explain the composition of vaginal metabolites. The visual display of these data as represented by the distance-based redundancy analysis (dbRDA) plot is displayed in Fig. 3.

Further, 142 metabolites had abundances that were marginally significantly different between smokers and non-smokers (q-value between 0.05 and 0.10) without adjustment for CST (Table S2). These compounds are involved in amino acid metabolism, including some essential for bodily functions such as lysine, tryptophan, leucine, isoleucine, histidine and methionine as well as many amino acid dipeptides required for protein hydrolysis. Interestingly, the greatest differences in the composition and abundance of the metabolomes between smokers and non-smokers were among biogenic amines, including, cadaverine (fold change in non-smokers, FC = −53.19), putrescine (FC = −15.80), agmatine (FC = −15.49), tyramine (FC = −5.15) and tryptamine (FC = −4.43), which were much higher in smokers (Table S2, Figure S2).

The associations observed between smoking and the vaginal metabolome may be explained in part by participant-specific variables, such as sexual behaviors, health, race and other confounding factors. We utilized a distance-based linear modeling (DISTLM) approach to explain the contribution of confounding participant variables to the vaginal metabolome (Table 1). Due to the relatively small sample size of our study, the model was limited to a total of four variables, which we selected as the best fit from all potential models with the inclusion of smoking status and CST. The model including CST, race, education level, and smoking status, had an adjustedR^2^ of 0.26, and these variables explained 34% of the observed variation in the vaginal metabolomic profiles (Table 1). Using this analytical approach based on the full metabolome, smoking status did not show a statistically significant relationship to the full spectrum of vaginal metabolites after adjustment for CST, race, and education, although in some cases, when the associations are large and the p-values do not reach significance, it may be due to the lack of power associated with the relatively small sample size **(**Fig. 3, Table 1).

**Figure 4:**
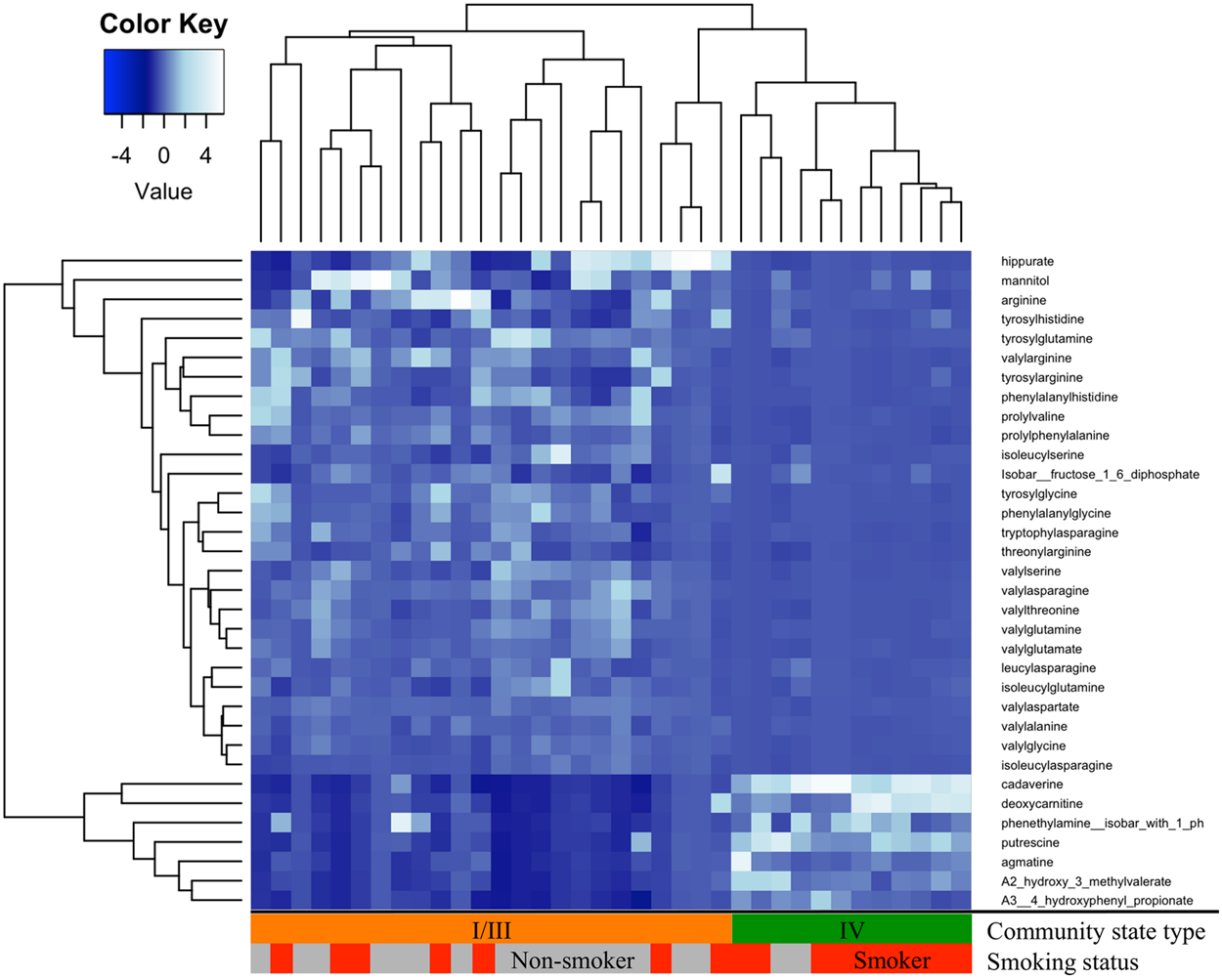
Metabolites differ between CST. Heatmap displays selection of metabolites with a high fold change identified as significantly (q-value = <0.05) different in the vagina of bacterial community state type (CST) when unadjusted and adjusted for the impact of smoking status. Low-*Lactobacillus* CST-IV and *Lactobacillus*-dominated CST-I and CST-III are grouped. Quantile regression was conducted on centered and scaled metabolite concentrations. Significance testing was conducted with Wilcoxon rank sum test and corrected for multiple comparisons.

In addition to the multivariate analyses above, we also sought to directly contrast the metabolome of *Lactobacillus*-dominated CST-I/III and the low-*Lactobacillus/*diverse anaerobes observed in CST-IV. To increase the power of significance testing, we combined *L*. *crispatus*-dominated CST-I and *L*. *iners*-dominated CST-III. In bivariate analysis, there was a significant difference (q-value < = 0.05) in the concentrations of 67% of all identified vaginal metabolites between CST-I/III and CST-IV (Table S3). After adjusting for the influence of a woman being a current smoker, the abundance of 54% of vaginal metabolites still differed significantly (q-value < = 0.05). The majority of the metabolites that were elevated in the *Lactobacillus*-dominated CSTs were amino acids, lipids, peptides and especially dipeptides (Fig. 4). Carbohydrates, mannitol, lactate, xylulose and fucose were higher in the *Lactobacillus*-dominated CSTs compared to CST-IV (Table S3). The glycine conjugate of benzoic acid, hippurate, was also markedly higher (34-fold) in *Lactobacillus*-dominated communities. The BAs (cadaverine, putrescine, agmatine, tyramine, tryptamine) and the straight chain fatty acid deoxycarnitine were also observed to be significantly higher in CST-IV compared to *Lactobacillus*-dominant communities (Table S3). Correlations with pH measurement and BA concentrations were positive, yet lacked statistical significance (Table S4, Figure S3).

Further, among women in CST-IV, the BAs were markedly higher in smokers versus non-smokers (q-value: <0.05, Cadaverine (89-fold), Putrescine (26-fold), Agamatine (26-fold), Tyramine (10-fold) and Trypatamine (7-fold), Figure S2).

## Discussion

Our group and others have previously reported that female smokers are more likely to display a low-*Lactobacillus* CST-IV vaginal microbiota and are at increased risk for morbidities such as bacterial vaginosis^9,30^ and sexually transmitted infections^39^. To obtain greater insight into this relationship, we assessed the vaginal metabolome of smokers and non-smokers. Using a comprehensive metabolomics approach and multiple analyses of the data, we identified an extensive and diverse range of vaginal metabolites for which profiles were affected by both the microbiology and smoking status. Bacterial composition (CST) was the most pronounced driver of the vaginal metabolome in our model and was associated with changes in 57% of all metabolites, suggesting vaginal microbiota are the major drivers of the vaginal metabolome. We therefore carefully controlled for CST in multivariate analysis and also conducted stratified analysis based on CST so that we could directly contrast smokers versus non-smokers, while essentially holding CST constant. As expected, nicotine and its breakdown products were markedly elevated in the vagina of smokers. In humans, 70–80% of nicotine is converted to cotinine followed by conversion to hydroxycotinine^58^. Hydroxycotinine is a well-known biomarker identified in the urine, plasma and serum of individuals exposed to both active and passive smoking^59–61^. Previous studies have identified nicotine and cotinine in the cervical mucus of smokers^27^ with good correlation to concentrations in blood and urine^62,63^. Our findings are consistent with the vaginal metabolome contributing to the mechanism linking smoking to the microbiome.

Another key finding was that we observed significant increases in the abundance of various BAs among smokers relative to non-smokers, which was far more pronounced in women with a low-*Lactobacillus* CST-IV. BAs are unique molecules that carry one or more amine groups (NH_2_). They are essential to mammalian and bacterial physiology, tightly controlled in cellular metabolisms. Cadaverine, putrescine, agmatine and tryptamine have roles in the metabolism of essential amino acids, including tryptophan and lysine. Their accumulation indicates upheaval or alterations in these metabolic systems. In particular, the odors of the amines cadaverine and putrescine are foul-smelling to humans, identifiable as indicators of tissue decomposition associated with death or bacterial contamination^64,65^. Several of these BAs, including cadaverine and putrescine, have previously been correlated with diagnosis of BV^45,66,67^ and implicated in the associated ‘fishy’ vaginal malodor of BV^46,47^.

Our group recently suggested a hypothetical model for the displacement of vaginal *Lactobacillus* spp. and increased risk of BV and urogenital infection with BAs^68^. We hypothesize that in the vaginal canal, BAs may favor non*-Lactobacillus* species, while also increasing the vaginal pH, collectively enabling colonization by a more diverse community, as is observed with CST-IV. Our hypothesis is based on two observations. First, the amino-acid decarboxylation reactions that produce BAs involve the consumption of intracellular hydrogen ions and is a well-described bacterial acid resistance and mitigation mechanism^52^. The consumption of hydrogen ions increases the pH of the local habitat^52^, overcoming what is widely considered the primary barrier to pathogen outgrowth and a clinically-recognized symptom of BV. Second, the growth of several pathogens, including the urogenital pathogen, *Neisseria gonorrhoeae*, and their resistance to host immunological defenses has been shown to be superior in the presence of various BAs^53–55,69^. In this study, we observed that lactate was lower and BAs were higher, in low-*Lactobacillus* CST-IV participants as expected. However, lactate was higher in *Lactobacillus*-dominated CST-I/III even when BA concentrations were low. Lactate concentration includes both protonated lactic acid (LAH) and lactate anion (LA^−^) with the former being recognized as the active microbicidal form capable of inactivating BV-associated bacteria^31,70^. Lactate concentrations increase with increasing hydrogen ion concentrations^31^ and therefore the reduction in lactate and increase in BAs further supports our hypotheses, and suggest that bacteria present in low-*Lactobacillus* CST-IV use available hydrogen ions to perform amino acid decarboxylation and produce BAs, thus resisting acid stress^56^.

Our results identifying the presence of nicotine breakdown products in smokers may reflect differences in the degree of transport and/or accumulation of nicotine and its derivatives in the vagina by CST. These differences may relate to the co-variation in pH with CST^33^. Absorption of nicotine across biological membranes (such as cervicovaginal endothelial/epithelial cells) has previously been shown to be pH dependent^56,71^. Nicotine is a weak base (pK_a_ = 8.0) and in acidic environments nicotine does not rapidly cross membranes^68^. Cotinine and hydroxycotinine are more acidic with a pK_a_ = 4.8 and 4.3, respectively^72^. Previous studies which have measured both in serum and vaginal samples identified higher levels of nicotine in the cervical mucus compared with serum samples, yet cotinine values were similar^27,63^. Nicotine may be selectively concentrated in the vagina because the majority (72%) of women display a vaginal pH of 4.0 to 4.6^33^. Conversely, women with low-*Lactobacillus* CST-IV vaginal microbiota have a vaginal pH of 5.3 ± 0.6^33^ and, consistently displayed comparatively lower levels of nicotine. Alternatively, these findings may indicate microbiological metabolism of nicotine in the more diverse and low-*Lactobacillus* state of CST-IV.

Similarly, hippurate, a normal excretory product of urine that is increased with exposure to phenolic compounds and toluene, a byproduct of cigarette smoke^73^, was increased in *Lactobacillus*-dominated CST-I/III over CST-IV and also in non-smokers over smokers. Hippurate is a known substrate of *Gardnerella vaginalis*^74^, an organism often found in higher abundances when *Lactobacillus* spp. are low and commonly associated with symptomatic BV^75–77^. Hippurate is decreased in the low-*Lactobacillus* CST-IV and this may suggest that microbial utilization of hippurate and therefore smoking may move the vaginal environment closer to favor the proliferation of *G*. *vaginalis*.

Beyond metabolites directly affiliated with nicotine metabolism, we observed substantive shifts in dipeptides and biogenic amines. More than 150 dipeptides in smokers or CST-IV participants were significantly decreased relative to non-smokers and those with *Lactobacillus* spp.-dominated vaginal microbiota. In a previous study, Ghartey and colleagues noted significant reductions in vaginal dipeptides among women who delivered infants preterm, for which CST-IV and BV are important risk factors^78^. Dipeptides refer to one or more amino acid joined by a peptide bond with important roles in protein metabolism and cell signaling. CST-I and III women had higher concentrations of dipeptides relative to CST-IV, suggesting *Lactobacillus* spp. dominance may be important to this phenotype. Dipeptides are constituents of the peptidoglycan cell wall of bacteria and are made during its’ synthesis. Therefore, some dipeptides, such as muramyl dipeptides, serve as signal to the immune system in mammals and play a direct role in the regulation of inflammation^79^. Their production has also been indicated in *Lactobacillus* spp. and other bacteria as a mean of quorum sensing and cellular signaling^80^. *Lactobacillus* spp. may exploit dipeptides to increase their osmotolerance^81^, or produce dipeptides for amensalistic purposes. Various *Lactobacillus* spp. have been noted to produce cyclic dipeptides with anti-fungal^80,82–84^, anti-viral^85^ and anti-bacterial^80,86^ properties. This includes the production of dipeptides by a vaginal isolate of *L*. *reuteri* that disrupts the virulence capabilities of *Staphylococcus aureus* involved in toxic shock syndrome^67^. The increase in dipeptides in non-smokers and *Lactobacillus* spp.-dominant CSTs may be reflective of increased production by *Lactobacillus* spp. of these bioactive compounds. Conversely, the reduction in dipeptides in women with a low-*Lactobacillus* CST-IV vaginal microbiota may be a result of the increased proteolytic activity from bacteria present in this CST. Many BV-associated bacteria have been correlated with, or shown to be capable of secreting proteases, which break down proteins into amino acids^87–91^. Aside from reducing the number of detected dipeptides in the vaginal tract, proteases may also have the effect of inactivating proteins important to host defenses and make host tissues more susceptible to other organsims’ virulence factors^87^.

The breakdown compounds of a number of drugs, such as cocaine (norbenzoylecgonine, benzoylecogonine), antidepressants (escitalopram), common painkillers (acetaminophen glucoronide, ibuprofen) and decongestants (pseudoephedrine) were each observed in one or more samples in the study. This indicates that some drugs could possibly be assessed from vaginal metabolomic profiles. There is relatively little known about the relationship between individual drugs and their impact on the vaginal microbiome. Cocaine use has been associated with shifts in bacterial phyla in the gut^92^ and additionally has been associated with a greater likelihood of contracting sexually transmitted infections^93,94^. The use of antidepressants has been linked to menstrual disorders and hormonal changes in women^95,96^ both of which may cause indirect shifts in the vaginal microbiota. As these studies may suggest a potential impact of drugs and medications and their byproducts on the vaginal microbiota, further exploration is needed to make any conclusions.

Our data are consistent with recent studies of the vaginal metabolome^45,47,97^. In prior studies, the BAs (cadaverine, putrescine and tyramine) were consistently higher in women with BV^45,47,48,97^. We did not detect trimethlyamine in our samples as has previously been identified and we suspect this is due to its high volatility^45,47,48,97^. Srinivasan *et al*. reported the same issue with their lack of trimethylamine detection using similar methods yet reported lower levels of trimethylamine oxide (an intermediary product) in women with BV using separate methods^97^. Therefore, there are limitations to the use of metabolomics, including particle resolution, compound sensitivity, polarity and volatility^98^. Identification of detected metabolites is further constrained by comparison to facility-built databases^98,99^. However, this is best achieved by utilizing standardized collection, preparation and^98^ pairing liquid chromatography (LC), gas chromatography (GC) and mass spectrometry (MS) which can enhance identification of analytes with differing characteristics^98^.

Aside from biogenic amines, McMillian *et al*. reported alpha-hydroxyisovalerate and gamma-hydroxybutyrate (GHB) as associated with BV and high bacterial diversity^47^. We also observed higher levels of alpha-hydroxyisovalerate and GHB in low-*Lactobacillus* CST-IV and additionally we observed a trend towards increases in smokers. Alpha-hydroxyisovalerate was positively correlated with diverse BV-associated bacteria such as *Atopobium, G*. *vaginalis, Dialister* and *Gemella*. McMillian *et al*. went on to show how *G*. *vaginalis*, a vaginal bacterial species commonly associated with BV is a producer of GHB^47^. Srinivasan *et al*. also identified 12-hydroxyeicosatetraenoic acid (12-HETE) in cases of BV and we also detected a significantly high abundance of this compound in women from CST-IV over the *Lactobacillus*-dominant CSTs (I or III), although it was not associated with smoking status^97^.

A limitation of our study was the small sample size and the distribution of smokers and non-smokers within each community state type (17 CST-I, 7 CST-III, 12 CST-IV participants). As a result, we have relatively reduced power in some of these statistical tests in stratified analyses. Some of the assumptions of traditional parametric statistical tests are sensitive to small sample sizes, and the use of non-parametric statistical tests, as was employed here, can overcome these assumptions with increased robustness to skewness and outliers. The use of a false discovery rate provided a further method for conservative interpretation of these results. We combined *L*. *crispatus*-dominated CST-I and *L*. *iners*-dominated CST-III because in this study, succinate was the only metabolite with abundance significantly different between samples belonging to CST-I compared to CST-III. Combining the two CSTs allowed us to increase our statistical power by comparing CST-I/III to CST-IV, however combining the *L*. *crispatus* and *L*.*iners*-dominated communities may not be functionally optimal. There is a growing body of research focused on *L*. *iners*^32,100–103^ that aims to evaluate its protective value as part of the vaginal microbiota. *L*. *iners* is commonly detected in healthy women, and interestingly in women with BV, as well as being among the first *Lactobacillus* spp. to recover after antibiotic treatment for BV^104^. Further, it is often considered to be a tipping point for some women at risk for BV^104^. Despite the small sample size, this study is unique in its use of metabolomics and microbial abundance data in combination with extensive participant behavioral data and its rigorous evaluation of smoking status by using quantification of carbon monoxide exhaled and cotinine in saliva. The depth of the analyses performed in this study, and the results obtained, may inform where to focus resources in a larger confirmatory study.

## Conclusions

It is well-documented that a vaginal microbiota dominated by *Lactobacillus* spp. is associated with reduced risk for urogenital infections, including sexually transmitted infections (STIs) and urinary tract (UTIs) infections^105^. In this study, we determined that overall smoking did not affect the vaginal metabolome after controlling for CST, but several key metabolites were elevated in smokers. Among women who were smokers and depauperate for *Lactobacillus* spp. (classified as CST-IV), we observed that they had significantly more perturbed metabolic profile than other CSTs when compared to their non-smoking counterparts. Biogenic amines were elevated in smokers and these metabolites have known roles in anaerobic bacterial proliferation, immune- and stress-resistance with a significant link to the development of BV, and possibly other reproductive tract infections. Women who smoke may have increased susceptibility to reproductive tract infections due to the observed increase in concentrations of BAs and the finding was even more pronounced among women who had low levels of *Lactobacillus* spp.

The metabolite profile of the vagina was strongly influenced by the resident microbiota as well as cigarette smoking in epidemiologic analyses that controlled for possible confounders. Detection of nicotine and its breakdown products in the vagina may serve as molecular biomarkers of smoking. Our results suggest that smoking is associated with several important metabolites present in the vagina that may have implications for women’s health. This study serves as a pilot for the development of future studies of the mechanisms linking smoking to poor gynecologic and reproductive health outcomes.

## Methods

### Sample Collection

Forty women self-collected mid-vaginal swabs during a single visit at the Center for Health Behavior Research at the University of Maryland School of Public Health (UMSPH). The study has previously been described^30^. In brief, smoking burden was determined by saliva cotinine testing, carbon monoxide exhalation levels and self-report of smoking habits on a set of comprehensive behavioral surveys. Participants were excluded if they had used an antibiotic or antimycotic in the 30 days prior. Four women were excluded from this analysis due to poor DNA quality affecting the normalization of the vaginal metabolome dataset (final sample size for analysis was 36 (17 smokers and 19 non-smokers) (Table 2). All participants provided written informed consent, and ethical approval was obtained from the Institutional Review Boards of the University of Maryland Baltimore (UMB), Montana State University and the UMSPH. All samples were collected and analysed in accordance with the relevant guidelines and regulations.

**Table 2:** Factors associated with smoking status, Baltimore, MD (n=36)

	Non-smoker		Smoker		p-value^1^
	n	%	n	%	
Participant details					
Age					0.011
19–28	16	44	4	11	
29–38	1	3	6	17	
39–48	2	6	7	19	
Marital status					0.066
Single, never married	17	47	8	22	
Separated, divorce, widowed	1	3	5	14	
Married	1	3	3	8	
Race					0.170
Asian/Pacific Islander	3	8	1	3	
White	8	22	4	11	
African American/black	4	11	10	28	
Hispanic	3	8	0	0	
Multi-racial	1	3	1	3	
Other	0	0	1	3	
Education level					1.000
High School, up to 12 years	0	0	1	3	
College and graduate, > 12 years	19	53	16	44	
**Clinical**					
CST					0.011
I, *L. crispatus*-dominated	13	36	4	11	
III, *L. iners*-dominated	4	11	3	8	
IV, Low-*Lactobacillus* spp.	2	6	10	28	
Nugent’s Gram stain score					0.000
0–3	17	47	8	22	
4–6	2	6	2	6	
7–9	0	0	7	19	
Vaginal pH					0.004
<=4.0	10	28	4	11	
4.1–5.5	1	5	1	3	
4.6–5.0	4	11	2	6	
>=5.1	4	11	10	28	
**Self-reported symptoms**					
Vaginal odor					0.969
No	15	42	16	44	
Yes	4	11	1	3	
Vaginal irritation, 24 hours prior					1.000
No	19	53	17	47	
Yes	0	0	0	0	
Vaginal itching, 24 hours prior					1.000
No	19	53	17	47	
Yes	0	0	0	0	
Vaginal burning, 24 hours prior					1.000
No	19	53	17	47	
Yes	0	0	0	0	
Pain urinating, 24 hours prior					1.000
No	19	53	17	47	
Yes	0	0	0	0	
Vaginal discharge, 24 hours prior					0.847
No	14	39	14	39	
Yes	5	14	3	8	
**Hygiene**					
Vaginal douche, 2 months prior					0.108
No	18	50	9	25	
Yes	1	3	7	19	
Not recorded	0	0	1	3	
Menstruating currently					0.075
No	9	25	9	25	
Yes	10	28	8	22	
Tampon or pad, 24 hours prior					0.456
No pad, no tampon	10	28	10	28	
Pad only	2	6	1	3	
Tampon only	4	11	2	6	
Tampon and pad	2	6	1	3	
Not recorded	1	3	3	8	
**Sexual behaviors**					
Lifetime number of sexual partners					0.25
0–6	13	36	3	8	
7+	6	17	12	33	
Not recorded	0	0	2	6	
Number of sex partners, 2 months prior					0.000
0	4	11	2	6	
1	12	33	14	39	
2	3	8	1	3	
Vaginal intercourse with a condom, 24 hours prior					0.673
No vaginal intercourse	14	39	9	25	
Vaginal intercourse no condom	3	8	6	17	
Vaginal intercourse with condom	2	6	2	6	
Anal intercourse, 24 hours prior					1.000
No	19	53	17	47	
Yes	0	0	0	0	
Sex toy use, 24 hours prior					1.000
No	19	53	17	47	
Yes	0	0	0	0	
Lubricant use, 24 hours prior					1.000
No	19	53	17	47	
Yes	0	0	0	0	
Partner type					0.000
Regular	10	28	12	33	
Occasional	2	6	0	0	
New	1	3	0	0	
Not recorded	6	17	5	14	
Receptive oral sex, 24 hours prior					0.543
Yes	2	6	1	3	
No	17	47	16	44	
Digital penetration, 24 hours prior					0.555
Yes	3	8	2	6	
No	16	44	15	42	
Thong use, 24 hours prior					0.000
Yes	6	17	3	8	
No	13	36	13	36	
Not recorded	0	0	1	3	

^1^p-value determined using Fisher’s exact test.

### Sample Preparation for Metabolomics

Vaginal samples were eluted from swabs (Starplex rayon swab) in 200 µl phosphate buffered saline (PBS) and subjected to both gas chromatography mass spectrometry (GC-MS) and liquid chromatography mass spectrometry (LC-MS) with Orbitrap Elite accurate mass platforms (Thermo Scientific, Waltham, MA, USA). Sample processing and analysis was performed by Metabolon (Durham, NC, USA) using an automated MicroLab STAR® system (Hamilton Company, Reno, NV, USA). Recovery standards were added prior to the first step in the extraction process for quality control purposes. Sample preparation was conducted using a proprietary series of organic and aqueous extractions to remove the protein fraction while allowing maximum recovery of small molecules. The resulting extract was divided into two fractions: one for analysis by LC and one for analysis by GC. Samples were placed briefly on a TurboVap® (Zymark, Hopkinton, MA, USA) to remove the organic solvent. Each sample was then frozen and dried under vacuum. Samples were then prepared for the appropriate instrument, either LC-MS or GC-MS.

### Liquid Chromatography and Gas Chromatography Mass Spectrometry

LC-MS measurements were conducted on a Waters ACQUITY ultra-performance liquid chromatograph (UPLC) and a ThermoFisher Scientific Orbitrap Elite high resolution/accurate mass spectrometer (Thermo Scientific, Waltham, MA, USA), which consisted of a heated electrospray ionization (HESI) source and orbitrap mass analyzer operated at a resolution 30,000 mass. The sample extract was dried then reconstituted in LC-compatible solvents, each of which contained eight or more injection standards at fixed concentrations to ensure injection and chromatographic consistency. One aliquot was analyzed using acidic positive ion optimized conditions and the other using basic negative ion optimized conditions in two independent injections using separate dedicated columns. Extracts reconstituted in acidic conditions were gradient eluted using water and methanol containing 0.1% formic acid, while the basic extracts, which also used water/methanol, contained 6.5 mM ammonium bicarbonate. The MS analysis alternated between MS and data-dependent MS^2^ scans using dynamic exclusion.

Samples for GC-MS analysis were re-dried under vacuum desiccation for a minimum of 24 hours prior to being derivatized under dried nitrogen using bistrimethyl-silyl-triflouroacetamide (BSTFA). The GC column was 5% phenyl and the temperature ramp is from 40° to 300 °C in a 16 min period. Samples were analyzed on a Thermo-Finnigan Trace DSQ fast-scanning single-quadrupole mass spectrometer using electron impact ionization. The instrument was tuned and calibrated for mass resolution and mass accuracy prior to use. Peaks in the GC-MS and LC-MS data were identified using Metabolon’s proprietary peak integration software to resolve sample metabolite peaks over noise. Complete details of methods describing metabolomic profiling are described in Lawton *et al*. (2008)^106^.

### Compound Identification and Preparation

Spectra corresponding to each metabolite were identified by comparison to library entries of purified metabolite standards and their distinction from more than 1,000 other commercially available purified standard compounds. The combination of chromatographic properties and mass spectra gave an indication of a match to the specific biogenic amine compound or an isobaric entity. Results were manually curated to ensure that data were accurate and to remove any system artifacts, miss assignments, and background noise.

### Taxonomic Assignment and Community State Type Profiling

DNA extraction, PCR amplification and sequencing of 16S rRNA gene amplicons from the vaginal tract of study participants were conducted in a prior study^30^. Briefly, the V1-V3 region of the 16S rRNA gene was PCR amplified using the primers 27F-YM + 3^107^ and 534R^57^ and pyrosequenced using a Roche 454 FLX instrument. Species level assignments of *Lactobacillus* were performed using higher order Markov Chain models using the software speciateIT (speciateIT.sourceforge.net)^33^. For each sample, community state types (CSTs) were assigned to individual samples based on diversity and relative abundances of different phylotypes as defined in the work of Gajer and Brotman *et al*.^108^. The vaginal microbiota samples were categorized into CST-I (*L*. *crispatus*-dominated), CST-III (*L*. *iners*-dominated) and CST-IV (low-*Lactobacillus*/high strict and facultative anaerobes) (Table 2) Two other well-documented CSTs, CST-II (*L*. *gasseri*-dominated) and CST-V (*L*. *jensenii*-dominated) were not identified in this limited sample and are less commonly found even in larger surveys of women^33^.

### Data Analyses

The metabolomic dataset was normalized to DNA concentration and missing values were imputed with the minimum detected value for that compound which essentially assigns values based on the sensitivity limit^109^. Data were then centered and scaled and the median was set equal to 1 prior to log transformation. Samples were visualized using a principal components analysis (PCA) (Figure S1). We performed quantile regression to estimate the median metabolite concentration differences 1) between smokers and non-smokers, 2) between CSTs and, 3) also with adjustment for confounding factors. P-values were obtained using the Wilcoxon rank-sum test. To correct for multiple tests, false discovery rate (FDR) and q-values were calculated for each compound where p = <0.05. Where a p-value estimates the proportion of all tests which will result in false positives (i.e. 5% where p-value = 0.05), a q-value estimates the proportion of significant tests that will result in false positives^110^. Initial investigations between *Lactobacillus*-dominated CST-I and CST-III yielded only one significant difference in the metabolite succinate (q-value = 0.009) with adjustment for smoking status. This metabolite was not identified as differing significantly (q-value = 0.05) in all further analyses, and therefore we grouped CST-I and CST-III for further binary analyses to increase power in comparisons to CST-IV. Fold change (FC) was calculated based on Guo *et al*.^111^ where *FC*_*i*_* = x*_*i*_ *- y*_*i*_ where *i* is metabolite of interest for the mean value of the control (*x*) and treatment (*y*).

We performed distance based linear modeling (DISTLM) by partitioning the distances of compounds using distance-based redundancy analysis (dbRDA)^112^, a form of multivariate multiple regression that can be performed directly on a distance or dissimilarity matrix. As the number of participants limits the number of variables that can be included in the model, we planned to include just two variables, in addition to our main factors of CST and smoking status. Initially we ran individual variables against the metabolite data and selected only those variables that were significant (p < = 0.05). These were age, race, marital status, education level, thong undergarment in the prior 24 hours, and vaginal douching in the two months prior. The dissimilarity matrix of vaginal metabolites was fitted with four of the variables listed in Table 2 and the final model was selected with the greatest adjusted R^2^ value.

Boxplots and heatmaps were constructed using the ggplot2^113^ and RColorBrewer (colorbrewer2.org) packages conducted in R version 3.1.2^114^. PCA, correlation coefficients, DISTLM and dbRDA plots were produced in Primer version 6^115^.

### Data availability

The questionnaire and metabolome data are available at the National Center for Biotechnology Information (NCBI) Database of Genotypes and Phenotypes (dbGaP) under Accession number phs001386.v1.p1. Metagenomic sequence data were submitted to the public NCBI Sequence Read Archive (SRA) with the accession number PRJNA391039.

## Electronic supplementary material

Supplementary Information
