# Supplementary material for: Cigarette smoking is associated with an altered vaginal tract metabolomic profile
Source: Sci Rep. 2018 Jan 16;8:852. doi: 10.1038/s41598-017-14943-3 (PMC5770521; doi:10.1038/s41598-017-14943-3)
Supplement: Supplementary file 1 — Supplementary Information [file 41598_2017_14943_MOESM1_ESM.docx]

### Title:

Cigarette smoking is associated with an altered vaginal tract metabolomic profile

### Authors:

Nelson TM^1,2^, Borgogna JC^1,2^, Michalek, RD^3^, Roberts DW^4^, Rath JM^5,6^, Glover ED^5^, Ravel J^7,8^, Shardell MD^9^, Yeoman CJ^1,2^, Brotman RM^7,10^

### Author Affiliations

^1^ Department of Animal and Range Sciences, Montana State University, Bozeman, MT, USA.

^2^ Department of Microbiology and Immunology, Montana State University, Bozeman, MT, USA.

^3^ Metabolon Inc., Durham, NC, USA.

^4^ Department of Ecology, Montana State University, Bozeman, MT, USA.

^5^ Department of Behavioral and Community Health, University of Maryland School of Public Health, College Park, MD, USA

^6^ Truth Initiative, Washington DC, USA

^7^ Institute for Genome Sciences, University of Maryland School of Medicine, Baltimore, MD, USA.

^8^ Department of Microbiology and Immunology, University of Maryland School of Medicine, Baltimore, MD, USA.

^9^ Translational Gerontology Branch, National Institute on Aging, Baltimore, MD, USA

^10^ Department of Epidemiology and Public Health, University of Maryland School of Medicine, Baltimore, MD, USA.

**Supplementary Material**

**Tables**

**Table S1. Significantly different vaginal metabolites between smokers and nonsmokers, unadjusted and adjusted for the bacterial community state type**

|  | **p-value** | **q-value** | | **Smoker mean** | **Non-smoker mean** | **FC in Non-smoker** |
| --- | --- | --- | --- | --- | --- | --- |
| **Smokers v Non-smokers, unadjusted where q-value <0.05** | | | | | | |
| Deoxycarnitine | 0.0002 | 0.0123 | 12.64 | | 0.62 | -12.02 |
| Nicotine | 0 | 0.0002 | 8.23 | | 0.22 | -8.01 |
| Tryptamine | 0.0004 | 0.0167 | 4.45 | | 0.02 | -4.43 |
| Xanthine | 0.0002 | 0.0123 | 5.09 | | 0.72 | -4.37 |
| 3-Methyl-2-oxovalerate | 0.001 | 0.0346 | 3.93 | | 0.68 | -3.25 |
| 3-Hydroxycotinine glucuronide | 0.0004 | 0.0167 | 3.28 | | 0.47 | -2.81 |
| Cotinine | 0 | 0 | 2.95 | | 0.18 | -2.77 |
| Uracil | 0.0002 | 0.0123 | 3.51 | | 0.8 | -2.71 |
| Alpha hydroxyisovalerate | 0.001 | 0.0346 | 2.5 | | 0.12 | -2.38 |
| Hydroxycotinine | 0 | 0 | 2.29 | | 0.44 | -1.85 |
| Adenosine-3-monophosphate (3-AMP) | 0.0015 | 0.046 | 0.88 | | 2.42 | 1.54 |
| Pyroglutamylvaline | 0.0017 | 0.0477 | 2.98 | | 4.95 | 1.97 |
| **Smokers v non-smokers, adjusted for community state type where q-value <0.05** | | | | | | |
| Hydroxycotinine | 0.0001 | 0.0000 |  | | | |
| Cotinine_vaginal | 0.0001 | 0.0000 |  |  |  |  |
| Nicotine | 0.0009 | 0.0000 |  |  |  |  |
| Adenosine-3-monophosphate (3-AMP) | 0.0111 | 0.0001 |  |  |  |  |
| 3-Hydroxycotinine_glucuronide | 0.0484 | 0.0004 |  |  |  |  |

Compounds identified as significantly different in the vagina of smokers and non-smokers. Quantile regression was conducted on centered and scaled metabolite concentrations. Significance testing was conducted with Wilcoxon rank sum test and corrected for multiple comparisons. Metabolites that differed significantly where q-value = <0.05 between smokers and non-smokers when unadjusted and adjusted for community state type. Mean value of metabolite concentrations are shown with standard deviation (SD). Fold change (FC) negative values indicate the decrease in non-smokers when compared with smokers.

**Table S2. Marginally significant (qvalue >=0.05 <0.10) different vaginal metabolites between smokers and nonsmokers, unadjusted for the bacterial community state type**

|  | **p-value** | **q-value** | | **Smoker mean** | **Non-smoker mean** | **FC in Non-smoker** |
| --- | --- | --- | --- | --- | --- | --- |
| **Smokers v Non-smokers, unadjusted where q-value >0.05 <0.10** | | | | | | |
| Cadaverine | 0.0310 | 0.0880 | 53.74 | | 0.55 | -53.19 |
| 3-4-Hydroxyphenyl-propionate | 0.0231 | 0.0839 | 17.30 | | 0.18 | -17.12 |
| Putrescine | 0.0252 | 0.0839 | 16.66 | | 0.86 | -15.80 |
| Agmatine | 0.0076 | 0.0771 | 16.06 | | 0.57 | -15.49 |
| 2-Hydroxy-3-methylvalerate | 0.0113 | 0.0771 | 12.92 | | 0.46 | -12.46 |
| Pipecolate | 0.0043 | 0.0771 | 7.01 | | 0.73 | -6.27 |
| Arginine | 0.0252 | 0.0839 | 16.54 | | 10.31 | -6.23 |
| Oleic ethanolamide | 0.0052 | 0.0771 | 6.54 | | 0.40 | -6.14 |
| 5-6-Dihydrothymine | 0.0110 | 0.0771 | 6.09 | | 0.86 | -5.22 |
| Tyramine | 0.0265 | 0.0839 | 6.48 | | 1.32 | -5.15 |
| Palmitoyl ethanolamide | 0.0335 | 0.0916 | 5.68 | | 0.93 | -4.75 |
| 5-Aminovalerate | 0.0056 | 0.0771 | 5.37 | | 0.64 | -4.73 |
| Stearoyl-ethanolamide | 0.0383 | 0.0932 | 5.58 | | 1.26 | -4.32 |
| Gamma-hydroxybutyrate (GHB) | 0.0039 | 0.0771 | 4.48 | | 0.89 | -3.58 |
| Succinate | 0.0337 | 0.0916 | 3.46 | | 1.04 | -2.42 |
| 2-Aminobutyrate | 0.0275 | 0.0839 | 3.35 | | 1.00 | -2.34 |
| Sorbitol | 0.0057 | 0.0771 | 4.42 | | 2.20 | -2.23 |
| Nicotinate | 0.0025 | 0.0635 | 2.78 | | 0.71 | -2.07 |
| N-Acetylputrescine | 0.0134 | 0.0771 | 3.93 | | 1.98 | -1.95 |
| 2-Hydroxybutyrate (AHB) | 0.0076 | 0.0771 | 2.36 | | 0.40 | -1.95 |
| 13-HODE-9-HODE | 0.0153 | 0.0771 | 2.59 | | 0.80 | -1.79 |
| 2-Hydroxyglutarate | 0.0449 | 0.0985 | 2.76 | | 1.07 | -1.69 |
| Sarcosine-N-methylglycine- | 0.0262 | 0.0839 | 1.74 | | 0.34 | -1.41 |
| Thymine | 0.0339 | 0.0916 | 1.92 | | 0.62 | -1.29 |
| Alpha-hydroxyisocaproate | 0.0252 | 0.0839 | 2.18 | | 0.88 | -1.29 |
| Serine | 0.0147 | 0.0771 | 4.58 | | 3.36 | -1.23 |
| Lignocerate-24-0 | 0.0163 | 0.0771 | 1.54 | | 0.54 | -1.00 |
| Malate | 0.0299 | 0.0858 | 1.81 | | 1.02 | -0.79 |
| Valylarginine | 0.0094 | 0.0771 | 12.03 | | 11.24 | -0.79 |
| Lathosterol | 0.0322 | 0.0907 | 1.65 | | 0.89 | -0.76 |
| Valylserine | 0.0134 | 0.0771 | 13.06 | | 12.56 | -0.50 |
| N-acetylthreonine | 0.0212 | 0.0807 | 1.97 | | 1.56 | -0.41 |
| prolylglutamate | 0.0275 | 0.0839 | 5.37 | | 4.98 | -0.39 |
| N-Acetylarginine | 0.0252 | 0.0839 | 2.00 | | 1.61 | -0.39 |
| Erucate (22:1n9) | 0.0325 | 0.0910 | 1.37 | | 1.02 | -0.36 |
| Prolylthreonine | 0.0094 | 0.0771 | 5.62 | | 5.54 | -0.09 |
| Valyllysine | 0.0355 | 0.0916 | 3.73 | | 3.65 | -0.08 |
| Adenosine-2-monophosphate-2-A | 0.0152 | 0.0771 | 1.28 | | 1.35 | 0.08 |
| Glycylproline | 0.0194 | 0.0802 | 3.05 | | 3.13 | 0.08 |
| Cis-4-decenoyl-carnitine | 0.0094 | 0.0771 | 1.59 | | 1.69 | 0.10 |
| N2-N2-Dimethylguanosine | 0.0143 | 0.0771 | 1.64 | | 1.74 | 0.10 |
| Cysteine | 0.0392 | 0.0932 | 2.42 | | 2.57 | 0.15 |
| 3-Methylcytidine | 0.0068 | 0.0771 | 2.21 | | 2.57 | 0.36 |
| Octanoylcarnitine | 0.0143 | 0.0771 | 2.36 | | 2.73 | 0.37 |
| Ribulose | 0.0122 | 0.0771 | 3.14 | | 3.55 | 0.40 |
| Prolylalanine | 0.0074 | 0.0771 | 5.20 | | 5.62 | 0.41 |
| Decanoylcarnitine | 0.0166 | 0.0771 | 2.41 | | 2.83 | 0.41 |
| Gamma-glutamylthreonine | 0.0288 | 0.0858 | 2.32 | | 2.76 | 0.44 |
| 1-Oleoylglycerophosphocholine | 0.0442 | 0.0985 | 0.93 | | 1.43 | 0.50 |
| Gamma-glutamyltryptophan | 0.0453 | 0.0987 | 2.11 | | 2.65 | 0.55 |
| 1-Stearoylglycerophosphoserine | 0.0035 | 0.0771 | 0.61 | | 1.16 | 0.56 |
| 1-Palmitoylplasmenylethanolamin | 0.0084 | 0.0771 | 0.47 | | 1.12 | 0.65 |
| Tyrosine | 0.0060 | 0.0771 | 2.90 | | 3.56 | 0.66 |
| 1-5-Anhydroglucitol-1-5 | 0.0264 | 0.0839 | 1.56 | | 2.27 | 0.71 |
| Prolylphenylalanine | 0.0177 | 0.0771 | 5.76 | | 6.55 | 0.79 |
| Tiglyl carnitine | 0.0064 | 0.0771 | 1.06 | | 1.87 | 0.80 |
| Phenylalanine | 0.0121 | 0.0771 | 2.40 | | 3.22 | 0.82 |
| Cytidine-5-monophosphate-5 | 0.0265 | 0.0839 | 1.19 | | 2.04 | 0.84 |
| Glutathione-oxidized (GSSG) | 0.0387 | 0.0932 | 1.30 | | 2.16 | 0.85 |
| Histidine | 0.0194 | 0.0802 | 1.99 | | 2.86 | 0.87 |
| Tigloylglycine | 0.0042 | 0.0771 | 0.60 | | 1.50 | 0.89 |
| Maltopentaose | 0.0420 | 0.0951 | 1.87 | | 2.77 | 0.90 |
| Phenylcarnitine | 0.0148 | 0.0771 | 1.93 | | 2.83 | 0.91 |
| Hexanoylcarnitine | 0.0082 | 0.0771 | 1.13 | | 2.05 | 0.91 |
| Gamma-glutamylphenylalanine | 0.0212 | 0.0807 | 2.22 | | 3.19 | 0.97 |
| Ophthalmate | 0.0364 | 0.0926 | 1.59 | | 2.59 | 1.00 |
| Isovalerylcarnitine | 0.0275 | 0.0839 | 2.86 | | 3.86 | 1.00 |
| Hydroxybutyrylcarnitine- | 0.0134 | 0.0771 | 0.98 | | 2.00 | 1.02 |
| Arabinose | 0.0392 | 0.0932 | 1.29 | | 2.32 | 1.03 |
| Glycyllysine | 0.0442 | 0.0985 | 2.61 | | 3.68 | 1.08 |
| Valylalanine | 0.0177 | 0.0771 | 6.10 | | 7.19 | 1.09 |
| 2-Oleoylglycerophosphocholine | 0.0392 | 0.0932 | 1.47 | | 2.61 | 1.14 |
| Xylulose | 0.0122 | 0.0771 | 2.85 | | 4.06 | 1.21 |
| Gamma-glutamylleucine | 0.0415 | 0.0948 | 3.00 | | 4.22 | 1.22 |
| Glutamate-gamma-methyl-ester | 0.0162 | 0.0771 | 0.77 | | 2.07 | 1.30 |
| 2-Methylbutyrylcarnitine-C5 | 0.0299 | 0.0858 | 2.07 | | 3.39 | 1.32 |
| Prolylglycine | 0.0299 | 0.0858 | 3.07 | | 4.39 | 1.32 |
| N-Acetylmethionine sulfoxide | 0.0161 | 0.0771 | 4.41 | | 5.77 | 1.35 |
| Threonine | 0.0231 | 0.0839 | 2.25 | | 3.61 | 1.36 |
| Squalene | 0.0208 | 0.0807 | 0.43 | | 1.80 | 1.37 |
| Prolyltyrosine | 0.0206 | 0.0807 | 4.83 | | 6.22 | 1.39 |
| Valerylcarnitine | 0.0159 | 0.0771 | 1.01 | | 2.41 | 1.40 |
| Valylasparagine | 0.0134 | 0.0771 | 10.62 | | 12.02 | 1.40 |
| Butyrylcarnitine | 0.0252 | 0.0839 | 2.41 | | 3.81 | 1.40 |
| Lysylvaline | 0.0161 | 0.0771 | 3.21 | | 4.64 | 1.43 |
| Prolylvaline | 0.0177 | 0.0771 | 6.65 | | 8.09 | 1.43 |
| Valylaspartate | 0.0299 | 0.0858 | 7.30 | | 8.77 | 1.46 |
| Urea | 0.0415 | 0.0948 | 4.96 | | 6.43 | 1.47 |
| Cysteine-glutathione disulfide | 0.0127 | 0.0771 | 1.48 | | 3.05 | 1.57 |
| Prolylglutamine | 0.0177 | 0.0771 | 3.07 | | 4.65 | 1.58 |
| O-Sulfo-L-tyrosine | 0.0339 | 0.0916 | 0.94 | | 2.54 | 1.60 |
| Gamma-glutamylglutamate | 0.0212 | 0.0807 | 2.23 | | 3.86 | 1.62 |
| Prolylmethionine | 0.0142 | 0.0771 | 2.98 | | 4.65 | 1.67 |
| Valylglycine | 0.0275 | 0.0839 | 6.18 | | 7.86 | 1.68 |
| Threonylvaline | 0.0147 | 0.0771 | 2.85 | | 4.57 | 1.72 |
| Isobutyrylcarnitine | 0.0058 | 0.0771 | 1.69 | | 3.52 | 1.83 |
| A4-hydroxyhippurate | 0.0275 | 0.0839 | 1.17 | | 3.03 | 1.86 |
| Valylglutamine | 0.0383 | 0.0932 | 9.02 | | 10.91 | 1.90 |
| Isoleucylarginine | 0.0212 | 0.0807 | 4.53 | | 6.66 | 2.13 |
| Arginylvaline | 0.0383 | 0.0932 | 2.21 | | 4.38 | 2.17 |
| Valylthreonine | 0.0353 | 0.0916 | 8.29 | | 10.47 | 2.19 |
| Phenylalanylproline | 0.0255 | 0.0839 | 2.04 | | 4.27 | 2.23 |
| Valylleucine | 0.0353 | 0.0916 | 5.08 | | 7.39 | 2.31 |
| Lysine | 0.0415 | 0.0948 | 2.78 | | 5.16 | 2.38 |
| Prolyltryptophan | 0.0133 | 0.0771 | 4.77 | | 7.34 | 2.57 |
| Threonylproline | 0.0177 | 0.0771 | 4.07 | | 6.66 | 2.59 |
| Tryptophylalanine | 0.0231 | 0.0839 | 2.67 | | 5.34 | 2.66 |
| Glutarylcarnitine-C5 | 0.0265 | 0.0839 | 2.79 | | 5.55 | 2.75 |
| 4-Methylcatechol sulfate | 0.0102 | 0.0771 | 1.54 | | 4.29 | 2.75 |
| Phenylalanylvaline | 0.0415 | 0.0948 | 2.14 | | 4.96 | 2.81 |
| N-Acetylneuraminate | 0.0353 | 0.0916 | 4.13 | | 6.97 | 2.84 |
| Threonylalanine | 0.0415 | 0.0948 | 2.26 | | 5.13 | 2.87 |
| Tryptophylphenylalanine | 0.0094 | 0.0771 | 2.67 | | 5.56 | 2.88 |
| Valylvaline | 0.0194 | 0.0802 | 3.86 | | 6.76 | 2.90 |
| Leucylglycine | 0.0415 | 0.0948 | 2.90 | | 5.88 | 2.97 |
| Lysylleucine | 0.0353 | 0.0916 | 2.74 | | 5.72 | 2.97 |
| Threonylleucine | 0.0100 | 0.0771 | 4.36 | | 7.37 | 3.01 |
| Valylphenylalanine | 0.0161 | 0.0771 | 4.37 | | 7.53 | 3.17 |
| Ribitol | 0.0382 | 0.0932 | 1.08 | | 4.44 | 3.36 |
| Valyltyrosine | 0.0161 | 0.0771 | 4.24 | | 7.60 | 3.36 |
| Lysylphenylalanine | 0.0449 | 0.0985 | 2.20 | | 5.62 | 3.41 |
| 3-Methylxanthine | 0.0264 | 0.0839 | 1.92 | | 5.48 | 3.56 |
| Aspartylproline | 0.0082 | 0.0771 | 2.75 | | 6.39 | 3.64 |
| Arginylproline | 0.0110 | 0.0771 | 4.32 | | 8.08 | 3.76 |
| Tryptophylasparagine | 0.0102 | 0.0771 | 6.55 | | 10.34 | 3.79 |
| Alanylphenylalanine | 0.0275 | 0.0839 | 2.73 | | 6.62 | 3.89 |
| N-Acetyl-cadaverine | 0.0358 | 0.0917 | 1.38 | | 5.46 | 4.08 |
| Lysyltyrosine | 0.0299 | 0.0858 | 2.92 | | 7.02 | 4.09 |
| Valyltryptophan | 0.0090 | 0.0771 | 3.37 | | 7.47 | 4.10 |
| Alanylleucine | 0.0449 | 0.0985 | 3.46 | | 7.61 | 4.15 |
| Serylleucine | 0.0212 | 0.0807 | 4.30 | | 8.52 | 4.22 |
| Serylphenyalanine | 0.0147 | 0.0771 | 2.92 | | 7.26 | 4.35 |
| 2-Oleoylglycerophosphoserine | 0.0284 | 0.0858 | 2.00 | | 6.40 | 4.40 |
| Threonylphenylalanine | 0.0121 | 0.0771 | 3.97 | | 8.44 | 4.48 |
| Threonylglutamate | 0.0383 | 0.0932 | 3.84 | | 8.35 | 4.51 |
| Ornithine | 0.0147 | 0.0771 | 1.97 | | 7.05 | 5.09 |
| Pro-hydroxy-pro | 0.0078 | 0.0771 | 2.28 | | 8.28 | 5.99 |
| Glutamine-leucine | 0.0252 | 0.0839 | 3.08 | | 9.16 | 6.08 |
| Tyrosylglycine | 0.0353 | 0.0916 | 5.92 | | 12.03 | 6.11 |
| Glutathione reduced (GSH) | 0.0039 | 0.0771 | 3.13 | | 9.44 | 6.30 |
| 1-Methylnicotinamide | 0.0190 | 0.0802 | 2.51 | | 10.49 | 7.98 |
| Hippurate | 0.0045 | 0.0771 | 5.00 | | 20.18 | 15.18 |

Compounds identified as trending towards significantly differing in the vagina of smokers and non-smokers (q-value >0.05 <0.10). Quantile regression was conducted on centered and scaled metabolite concentrations. Significance testing was conducted with Wilcoxon rank sum test and corrected for multiple comparisons. Mean value of metabolite concentrations are shown with standard deviation (SD). Fold change (FC) negative values indicate the decrease in non-smokers when compared with smokers

**Table S3. Vaginal metabolites differing between community state types after adjustment for smoking**

|  | **CST-IV Non-smoker** | ***Lactobacillus*-dominated Non-smoker** | **CST-IV Smoker** | ***Lactobacillus*-dominated Smoker** | **FC in Non-smokers** | **FC in *Lactobacillus*-dominated-CST** |
| --- | --- | --- | --- | --- | --- | --- |
| **Low-*Lactobacillus* CST-IV v *Lactobacillus*-dominated CST-I and CST-III, adjusted for smoking status where q-value <0.05** | | | | | | |
| Cadaverine | 1.73 | 0.41 | 91.22 | 0.2 | -89.28 | -92.34 |
| 3-4-Hydroxyphenyl propionate | 0.62 | 0.13 | 29.33 | 0.13 | -28.7 | -29.69 |
| Putrescine | 1.96 | 0.73 | 28.04 | 0.41 | -25.76 | -28.85 |
| Agmatine | 0.34 | 0.6 | 27 | 0.43 | -26.49 | -26.31 |
| 2-Hydroxy-3-methylvalerate | 1.21 | 0.37 | 21.66 | 0.43 | -20.51 | -22.06 |
| Deoxycarnitine | 0.57 | 0.62 | 20.71 | 1.13 | -20.64 | -19.53 |
| Phenethylaminen (isobar with 1-ph) | 0.42 | 1.5 | 21.14 | 1.09 | -20.31 | -18.98 |
| Pipecolate | 0.71 | 0.74 | 11.5 | 0.59 | -10.65 | -10.88 |
| Oleic ethanolamide | 0.29 | 0.41 | 10.77 | 0.49 | -10.56 | -10.16 |
| Mead acid | 3.64 | 0.78 | 7.23 | 0.86 | -3.66 | -9.22 |
| 5-Aminovalerate | 1.28 | 0.56 | 8.68 | 0.65 | -7.48 | -8.75 |
| Tryptamine | 0.02 | 0.02 | 7.54 | 0.02 | -7.52 | -7.52 |
| N-Acetylputrescine | 0.47 | 1.01 | 8.21 | 0.79 | -7.52 | -6.89 |
| Xanthine | 0.8 | 0.71 | 7.84 | 1.15 | -7.47 | -6.77 |
| Tyramine | 0.2 | 1.46 | 9.31 | 2.42 | -10.08 | -5.63 |
| Gamma-hydroxybutyrate (GHB) | 0.89 | 0.89 | 6.76 | 1.21 | -6.18 | -5.55 |
| Nicotinate | 2.77 | 0.47 | 3.95 | 1.12 | -1.82 | -5.14 |
| Alpha-hydroxyisovalerate | 0.45 | 0.08 | 4.19 | 0.09 | -3.75 | -4.47 |
| Uracil | 1.11 | 0.76 | 4.96 | 1.43 | -4.52 | -3.87 |
| Indolepropionate | 1.81 | 0.34 | 2.18 | 0.31 | -0.33 | -3.34 |
| Sorbitol | 0.32 | 2.42 | 6.65 | 1.24 | -5.15 | -3.32 |
| 2-Hydroxybutyrate (AHB) | 0.38 | 0.41 | 3.56 | 0.64 | -3.41 | -2.9 |
| 12-HETE | 2.44 | 0.92 | 2.22 | 1.45 | -0.31 | -2.28 |
| 2-Deoxyinosine | 0.23 | 0.55 | 2.85 | 0.29 | -2.36 | -2.24 |
| Alpha-hydroxyisocaproate | 1.03 | 0.87 | 3.01 | 0.99 | -2.1 | -2.18 |
| N-Palmitoyltaurine | 0.53 | 0.55 | 2.08 | 0.4 | -1.41 | -1.66 |
| 13-HODE | 0.95 | 0.78 | 3.1 | 1.86 | -3.23 | -1.41 |
| Adenosine-2-monophosphate-2 | 4.82 | 0.94 | 0.11 | 2.95 | 2.71 | -1.04 |
| 4-Hydroxyphenylacetate | 0.23 | 0.98 | 1.87 | 0.11 | -0.77 | -1.02 |
| 3-Phenylpropionate hydrocinnamic | 0.31 | 0.08 | 0.71 | 0.08 | -0.4 | -0.85 |
| 1-Stearoylglycerophosphocholine | 0.96 | 2.02 | 2.86 | 2.05 | -1.92 | 0.25 |
| Cytidine-5-monophosphate-5 | 2.1 | 1.88 | 0.49 | 1.58 | 1.9 | 0.87 |
| Choline phosphate | 2.07 | 1.43 | 0.67 | 2.67 | 0.16 | 1.37 |
| 4-Hydroxyphenylpyruvate | 0.09 | 0.92 | 0.17 | 0.94 | -0.1 | 1.6 |
| 2-Stearoylglycerophosphoglycerol* | 0.17 | 0.86 | 0.28 | 1.3 | -0.55 | 1.71 |
| Sphingosine | 0.28 | 1.8 | 1.45 | 1.93 | -1.3 | 2 |
| 1-Palmitoylplasmenylethanolamine | 0.05 | 1.25 | 0.13 | 0.96 | 0.21 | 2.02 |
| 1-Palmitoylglycerophosphocholine | 0.98 | 2.54 | 3.29 | 3.86 | -3.63 | 2.12 |
| Fucose | 0.3 | 1.68 | 0.74 | 1.78 | -0.54 | 2.42 |
| Myristoylcarnitine | 0.17 | 1.38 | 0.16 | 1.44 | -0.06 | 2.49 |
| Xanthosine | 0.26 | 1.16 | 0.35 | 2 | -0.92 | 2.55 |
| 1-Stearoylglycerophosphoserine | 0.05 | 1.29 | 0.05 | 1.4 | -0.11 | 2.6 |
| Phosphate | 0.33 | 1.84 | 1.04 | 2.3 | -1.19 | 2.77 |
| 1-Oleoylglycerophosphocholine | 0.12 | 1.59 | 0.36 | 1.75 | -0.4 | 2.86 |
| Trans-4-Hydroxyproline | 0.19 | 2.17 | 1.09 | 2 | -0.72 | 2.88 |
| Ethyl glucuronide | 0.04 | 1.43 | 0.27 | 1.92 | -0.72 | 3.04 |
| 1-Oleoylglycerol-1-monoolein | 0.44 | 2.05 | 0.73 | 2.19 | -0.43 | 3.07 |
| Creatinine | 0.06 | 1.89 | 0.89 | 2.15 | -1.1 | 3.09 |
| Xylose | 0.4 | 1.98 | 1.36 | 2.94 | -1.91 | 3.15 |
| Hydroxybutyrylcarnitine_ | 0.6 | 2.17 | 0.32 | 1.93 | 0.52 | 3.17 |
| Glutamate gamma-methyl-ester | 0.23 | 2.29 | 0.26 | 1.5 | 0.76 | 3.3 |
| Nicotinate ribonucleoside_ | 0.21 | 1.59 | 0.82 | 2.78 | -1.79 | 3.33 |
| Beta-Hydroxyisovaleroylcarnitine | 0.3 | 1.9 | 0.54 | 2.27 | -0.61 | 3.33 |
| Palmitoylcarnitine | 0.19 | 2.33 | 0.65 | 1.84 | 0.02 | 3.33 |
| 1-Palmitoylglycerophosphoethanol | 0.56 | 1.76 | 0.79 | 3.04 | -1.51 | 3.45 |
| Glycerol-3-phosphate | 0.51 | 1.97 | 0.84 | 2.92 | -1.28 | 3.54 |
| Gamma-Glutamylalanine | 0.53 | 1.92 | 0.72 | 2.92 | -1.19 | 3.58 |
| 1-Stearoylglycerophosphoethanol | 0.66 | 1.77 | 0.47 | 2.97 | -1 | 3.61 |
| Myo-inositol | 0.33 | 1.79 | 1.12 | 3.33 | -2.33 | 3.67 |
| Picolinate | 0.33 | 1.92 | 1.13 | 3.22 | -2.09 | 3.68 |
| S-Methylglutathione | 0.18 | 2.2 | 0.04 | 1.73 | 0.62 | 3.71 |
| O-Methylcatechol sulfate | 0.13 | 2.15 | 0.82 | 2.53 | -1.06 | 3.74 |
| Uridine | 0.13 | 1.85 | 0.55 | 2.59 | -1.16 | 3.76 |
| 2-Oleoylglycerol-2-monoolein | 0.36 | 2.52 | 0.62 | 2.22 | 0.04 | 3.77 |
| Cis-zeatin | 0.28 | 1.91 | 1.17 | 3.35 | -2.33 | 3.82 |
| 1-Palmitoylglycerophosphoglycer | 0.5 | 2.02 | 0.61 | 2.92 | -1 | 3.83 |
| Proline | 0.37 | 1.95 | 0.69 | 2.98 | -1.35 | 3.87 |
| Guanosine | 0.1 | 2.13 | 0.3 | 2.23 | -0.3 | 3.96 |
| Creatine | 0.45 | 2.02 | 0.61 | 3.01 | -1.15 | 3.98 |
| Leucyltyrosine | 0.38 | 3.79 | 1.21 | 1.81 | 1.15 | 4.01 |
| Tiglyl carnitine | 0.33 | 2.05 | 0.12 | 2.42 | -0.15 | 4.02 |
| Acetylcarnitine | 0.54 | 2.06 | 0.44 | 2.99 | -0.84 | 4.06 |
| 5-Hydroxymethylfurfural | 0.26 | 1.88 | 0.74 | 3.2 | -1.8 | 4.08 |
| Oxalate ethanedioate | 0.42 | 2.09 | 1.18 | 3.63 | -2.3 | 4.12 |
| Isoleucyltryptophan | 0.11 | 3.04 | 0.88 | 2.14 | 0.12 | 4.2 |
| Laurylcarnitine | 0.1 | 2.17 | 0.21 | 2.37 | -0.31 | 4.23 |
| Glutathione oxidized (GSSG) | 0.78 | 2.32 | 0.19 | 2.9 | 0.02 | 4.25 |
| Pseudouridine | 0.13 | 2.55 | 0.56 | 2.43 | -0.32 | 4.28 |
| 2-Palmitoleoylglycerophosphoeth | 0.5 | 2.33 | 0.82 | 3.29 | -1.28 | 4.3 |
| Hexanoylcarnitine | 0.48 | 2.23 | 0.05 | 2.68 | -0.01 | 4.38 |
| Nicotinamide riboside | 0.12 | 3.01 | 0.75 | 2.25 | 0.13 | 4.4 |
| Valerylcarnitine | 0.22 | 2.67 | 0.14 | 2.25 | 0.5 | 4.55 |
| Tyrosylphenylalanine | 0.13 | 2.29 | 0.33 | 2.77 | -0.68 | 4.6 |
| Erythronate | 0.17 | 2.38 | 0.85 | 3.28 | -1.58 | 4.64 |
| Cytidine-5-diphosphocholine | 0.58 | 2.95 | 0.55 | 2.85 | 0.12 | 4.67 |
| Cis-4-Decenoyl carnitine | 0.75 | 1.8 | 0.09 | 3.74 | -1.28 | 4.7 |
| N-Acetylarginine | 0.13 | 2.81 | 0.43 | 2.49 | 0.03 | 4.74 |
| Glycerophosphorylcholine (GPC) | 0.2 | 2.55 | 0.49 | 2.92 | -0.66 | 4.78 |
| Arabitol | 0.28 | 3.01 | 1.26 | 3.36 | -1.32 | 4.83 |
| Catechol sulfate | 0.09 | 2.8 | 0.63 | 2.75 | -0.49 | 4.84 |
| Tartarate | 0.47 | 2.27 | 1.1 | 4.14 | -2.5 | 4.85 |
| Gluconate | 0.2 | 2.32 | 0.58 | 3.3 | -1.36 | 4.85 |
| Carnitine | 0.59 | 2.19 | 0.62 | 3.93 | -1.77 | 4.92 |
| 1-Oleoylglycerophosphoserine | 0.66 | 2.64 | 0.12 | 3.11 | 0.07 | 4.97 |
| Tryptophyltryptophan | 0.1 | 2.67 | 0.33 | 2.77 | -0.33 | 5.01 |
| 2-Linoleoylglycerophosphocholine | 0.47 | 2.14 | 0.29 | 3.71 | -1.39 | 5.09 |
| 1-5-Anhydroglucitol | 0.58 | 2.47 | 0.25 | 3.44 | -0.64 | 5.09 |
| 1-Linoleoylglycerol-1-monolino | 0.37 | 2.88 | 0.71 | 3.31 | -0.77 | 5.1 |
| 4-Hydroxyhippurate | 0.05 | 3.38 | 0.44 | 2.22 | 0.77 | 5.11 |
| Nicotinamide | 0.6 | 2.12 | 0.7 | 4.3 | -2.29 | 5.12 |
| Taurine | 0.36 | 2.52 | 1.14 | 4.14 | -2.4 | 5.16 |
| Maltotriose | 1.17 | 3.1 | 0.71 | 3.97 | -0.42 | 5.19 |
| 2-Oleoylglycerophosphocholine | 0.6 | 2.85 | 0.23 | 3.25 | -0.03 | 5.27 |
| Alanylalanine | 0.14 | 3.17 | 0.76 | 3.1 | -0.55 | 5.36 |
| 3-Dehydrocarnitine_ | 0.19 | 2.33 | 0.44 | 3.72 | -1.64 | 5.43 |
| Phenylcarnitine_ | 0.22 | 3.14 | 0.85 | 3.47 | -0.95 | 5.54 |
| Ophthalmate | 0.54 | 2.83 | 0.22 | 3.54 | -0.4 | 5.61 |
| Pyruvate | 0.12 | 2.47 | 0.9 | 4.18 | -2.5 | 5.63 |
| Glutamine | 0.35 | 3.15 | 0.75 | 3.68 | -0.94 | 5.73 |
| Inosine | 0.26 | 1.75 | 0.25 | 4.51 | -2.75 | 5.75 |
| 1-Linoleoylglycerophosphoserine | 0.37 | 2.86 | 0.17 | 3.44 | -0.38 | 5.76 |
| Isoleucylleucine | 0.14 | 3.88 | 1.13 | 3.26 | -0.37 | 5.88 |
| Methionylglutamate | 0.44 | 3.87 | 0.6 | 3.1 | 0.61 | 5.92 |
| 2-Linoleoylglycerol-2-monolino | 0.24 | 3.52 | 0.53 | 3.23 | 0.01 | 5.98 |
| Gamma-Glutamyltyrosine | 0.41 | 2.96 | 0.68 | 4.28 | -1.59 | 6.14 |
| Paraxanthine | 0.25 | 1.76 | 0.67 | 5.31 | -3.97 | 6.16 |
| Seryltyrosine | 0.14 | 3.78 | 0.44 | 2.98 | 0.51 | 6.18 |
| Succinylcarnitine | 0.54 | 2.4 | 1.14 | 5.47 | -3.68 | 6.19 |
| Methionylglycine | 0.22 | 4.62 | 0.65 | 2.52 | 1.67 | 6.27 |
| Cysteine glutathione disulfide | 0.13 | 3.39 | 0.22 | 3.29 | 0.01 | 6.34 |
| Histidine | 0.33 | 3.16 | 0.5 | 4.12 | -1.12 | 6.45 |
| Histidylproline | 0.07 | 2.97 | 0.48 | 4.03 | -1.46 | 6.46 |
| Isoleucyltyrosine | 0.25 | 4.3 | 1.02 | 3.47 | 0.06 | 6.49 |
| 5-Acetylamino-6-formylamin-3-m | 0.1 | 2.66 | 0.99 | 4.93 | -3.16 | 6.5 |
| 1-7-Dimethylurate | 0.23 | 3.28 | 0.55 | 4.14 | -1.18 | 6.64 |
| Octanoylcarnitine | 1.78 | 2.84 | 0.06 | 5.64 | -1.08 | 6.64 |
| Theophylline | 0.53 | 2.02 | 0.58 | 5.8 | -3.83 | 6.71 |
| Gamma-Glutamyltryptophan | 0.34 | 2.92 | 0.37 | 4.59 | -1.69 | 6.8 |
| 4-Acetylcytidine | 0.15 | 3.96 | 0.46 | 3.46 | 0.2 | 6.81 |
| 5-Oxoproline | 0.22 | 3.21 | 0.68 | 4.55 | -1.79 | 6.86 |
| Isoleucine | 0.17 | 3.35 | 1.65 | 5.49 | -3.62 | 7.02 |
| 4-Methylcatechol sulfate | 0.11 | 4.78 | 0.55 | 2.95 | 1.4 | 7.08 |
| Gamma-glutamylthreonine_ | 0.32 | 3.05 | 0.5 | 4.92 | -2.05 | 7.14 |
| Histidylvaline | 0.39 | 4.96 | 0.66 | 3.22 | 1.47 | 7.14 |
| Prolylproline | 0.3 | 3 | 0.57 | 5.09 | -2.35 | 7.22 |
| Glycerophosphoethanolamine | 0.08 | 3.64 | 0.36 | 4.03 | -0.67 | 7.23 |
| Adenosine | 0.13 | 2.82 | 0.39 | 4.97 | -2.41 | 7.28 |
| Gamma-Glutamylphenylalanine | 0.36 | 3.53 | 0.52 | 4.65 | -1.29 | 7.29 |
| Tryptophan | 0.24 | 3.33 | 0.68 | 5.01 | -2.12 | 7.42 |
| Gamma-Glutamylglutamate | 0.74 | 4.22 | 0.61 | 4.55 | -0.2 | 7.42 |
| Lactate | 0.22 | 3.3 | 0.52 | 4.88 | -1.89 | 7.45 |
| Isobutyrylcarnitine | 0.28 | 3.9 | 0.12 | 3.94 | 0.12 | 7.45 |
| Maltopentaose | 0.03 | 3.1 | 0.03 | 4.5 | -1.4 | 7.53 |
| 1-Methylurate | 0.18 | 3.84 | 0.63 | 4.51 | -1.13 | 7.54 |
| Propionylcarnitine | 0.36 | 3.63 | 0.29 | 4.6 | -0.91 | 7.58 |
| Tyrosyltyrosine | 0.18 | 4.01 | 0.6 | 4.37 | -0.79 | 7.6 |
| Lysyltryptophan | 0.08 | 5.07 | 0.36 | 2.99 | 1.8 | 7.62 |
| Tryptophyltyrosine | 0.3 | 4.14 | 1.16 | 5.13 | -1.85 | 7.8 |
| Leucylaspartate | 0.4 | 5.61 | 0.93 | 3.52 | 1.57 | 7.8 |
| 3-Methylcytidine | 0.07 | 2.86 | 0.12 | 5.19 | -2.38 | 7.85 |
| Threonylmethionine | 0.05 | 4.41 | 0.44 | 3.93 | 0.1 | 7.86 |
| Decanoylcarnitine | 0.85 | 3.06 | 0.08 | 5.74 | -1.92 | 7.87 |
| Isoleucylisoleucine | 0.17 | 5.04 | 1.56 | 4.57 | -0.93 | 7.89 |
| 7-Methylurate | 0.23 | 5.1 | 0.62 | 3.63 | 1.08 | 7.89 |
| Cysteine | 0.19 | 2.85 | 0.25 | 5.51 | -2.73 | 7.92 |
| Leucylthreonine | 0.24 | 4.86 | 0.95 | 4.26 | -0.11 | 7.93 |
| Histidylleucine | 0.23 | 5.01 | 0.73 | 3.91 | 0.61 | 7.96 |
| Threonine | 0.25 | 4.01 | 0.5 | 4.74 | -0.99 | 8 |
| Leucine | 0.21 | 3.6 | 0.86 | 5.49 | -2.53 | 8.02 |
| Phenylalanine | 0.28 | 3.57 | 0.44 | 5.2 | -1.8 | 8.06 |
| 2-Methylbutyrylcarnitine | 0.28 | 3.75 | 0.16 | 4.79 | -0.91 | 8.09 |
| Alpha-Glutamyltyrosine | 0.49 | 6.62 | 0.95 | 2.91 | 3.25 | 8.09 |
| Phenylalanylproline | 0.16 | 4.76 | 0.6 | 4.11 | 0.22 | 8.1 |
| Glutamate | 0.33 | 5.07 | 0.68 | 4.05 | 0.68 | 8.11 |
| Alanyltryptophan | 0.2 | 5.2 | 0.8 | 3.95 | 0.64 | 8.14 |
| Phenylacetylglutamine | 0.13 | 4.83 | 0.8 | 4.28 | -0.11 | 8.17 |
| Valylhistidine | 0.05 | 3.79 | 0.6 | 5.15 | -1.91 | 8.29 |
| 7-Methylxanthine | 0.29 | 4.86 | 0.62 | 4.41 | 0.11 | 8.36 |
| Isoleucylphenylalanine | 0.25 | 5.33 | 1.11 | 4.5 | -0.03 | 8.48 |
| N-Acetylthreonine | 0.12 | 3.72 | 0.39 | 5.32 | -1.87 | 8.53 |
| Nicotinamide adenine dinucleotid | 0.04 | 2.7 | 0.5 | 6.41 | -4.16 | 8.57 |
| Valylisoleucine | 0.18 | 4.31 | 0.96 | 5.47 | -1.94 | 8.64 |
| Alanyltyrosine | 0.14 | 4.78 | 0.59 | 4.62 | -0.29 | 8.68 |
| Glycylglycine | 0.29 | 3.99 | 0.43 | 5.48 | -1.62 | 8.76 |
| Isoleucylglutamate | 0.17 | 5 | 0.73 | 4.73 | -0.29 | 8.83 |
| Butyrylcarnitine | 0.33 | 4.22 | 0.33 | 5.38 | -1.15 | 8.94 |
| Aspartylvaline | 0.52 | 7.29 | 0.76 | 2.99 | 4.06 | 8.99 |
| Aspartylphenylalanine | 0.3 | 6.9 | 0.79 | 3.33 | 3.08 | 9.13 |
| Asparagylleucine | 0.25 | 7.54 | 0.98 | 2.9 | 3.91 | 9.21 |
| Arginylvaline | 0.11 | 4.88 | 0.38 | 4.83 | -0.22 | 9.23 |
| 3-Methylxanthine | 0.38 | 6.08 | 0.45 | 4.02 | 1.99 | 9.28 |
| Alanylvaline | 0.13 | 5.27 | 0.73 | 4.93 | -0.27 | 9.33 |
| Alanylproline | 0.09 | 3.84 | 0.5 | 6.1 | -2.67 | 9.35 |
| Neopterin | 0.11 | 3.53 | 0.53 | 6.49 | -3.39 | 9.39 |
| Serylproline | 0.16 | 4.23 | 0.43 | 5.75 | -1.79 | 9.39 |
| Serylvaline | 0.28 | 5.55 | 0.77 | 4.94 | 0.12 | 9.43 |
| Methionine | 0.24 | 3.88 | 0.73 | 6.57 | -3.19 | 9.47 |
| Theobromine | 1.22 | 5.47 | 0.47 | 5.75 | 0.46 | 9.54 |
| Aspartyltryptophan | 0.21 | 6.74 | 0.67 | 3.68 | 2.6 | 9.55 |
| Serylisoleucine_ | 0.15 | 4.96 | 1.08 | 5.84 | -1.82 | 9.58 |
| Gamma-Glutamylleucine | 0.45 | 4.67 | 0.72 | 6.26 | -1.87 | 9.76 |
| Glycylproline | 0.19 | 3.48 | 0.39 | 6.86 | -3.58 | 9.77 |
| Tryptophylglutamate | 0.33 | 3.69 | 0.76 | 7.19 | -3.93 | 9.8 |
| Glycyllysine | 0.09 | 4.11 | 0.21 | 6.03 | -2.04 | 9.84 |
| 6-Carbamoylthreonyladenosine | 0.19 | 5.94 | 0.6 | 4.76 | 0.78 | 9.92 |
| Isovalerylcarnitine | 0.53 | 4.25 | 0.29 | 6.52 | -2.04 | 9.95 |
| Threonylalanine | 0.1 | 5.72 | 0.43 | 4.86 | 0.52 | 10.05 |
| Tyrosine | 0.25 | 3.95 | 0.26 | 6.68 | -2.74 | 10.12 |
| Tryptophylglycine | 0.27 | 5.48 | 0.63 | 5.57 | -0.45 | 10.15 |
| Lysylphenylalanine | 0.12 | 6.26 | 0.53 | 4.59 | 1.26 | 10.2 |
| Ribulose | 0.49 | 3.9 | 0.34 | 7.15 | -3.1 | 10.24 |
| Tyrosylleucine | 0.2 | 5.41 | 0.68 | 5.77 | -0.84 | 10.3 |
| Prolylarginine | 0.06 | 5.02 | 0.19 | 5.57 | -0.67 | 10.34 |
| Xylulose | 0.28 | 4.51 | 0.33 | 6.46 | -2 | 10.36 |
| Lysine | 0.62 | 5.69 | 0.59 | 5.92 | -0.19 | 10.4 |
| 2-Oleoylglycerophosphoserine | 0.71 | 7.07 | 0.33 | 4.39 | 3.06 | 10.42 |
| Threonylvaline | 0.17 | 5.09 | 0.51 | 6.2 | -1.45 | 10.61 |
| Tryptophylalanine | 0.34 | 5.93 | 0.55 | 5.71 | 0.01 | 10.74 |
| Gamma-Glutamylglutamine | 0.26 | 7.28 | 0.72 | 4.57 | 2.26 | 10.87 |
| Glycine | 0.38 | 4.07 | 0.72 | 7.98 | -4.25 | 10.95 |
| Prolylglycine | 0.27 | 4.88 | 0.44 | 6.83 | -2.12 | 11 |
| Aspartate | 0.18 | 4.95 | 0.75 | 7.05 | -2.68 | 11.07 |
| Adenosine-5-monophosphate (AMP) | 8.86 | 4.85 | 0.12 | 15.22 | -1.64 | 11.08 |
| Alpha-Glutamylglutamate | 0.23 | 8.1 | 0.63 | 4 | 3.7 | 11.23 |
| Lysylvaline | 0.28 | 5.15 | 0.52 | 7.05 | -2.15 | 11.41 |
| Leucylproline | 0.14 | 4.78 | 0.53 | 7.34 | -2.95 | 11.45 |
| Glycyltryptophan | 0.21 | 5.82 | 0.99 | 6.86 | -1.82 | 11.48 |
| Lysylleucine | 0.18 | 6.37 | 0.54 | 5.89 | 0.12 | 11.53 |
| Tryptophylphenylalanine | 0.16 | 6.19 | 0.33 | 6.03 | 0 | 11.73 |
| pyroglutamylvaline | 0.33 | 5.49 | 0.28 | 6.85 | -1.3 | 11.74 |
| Ornithine | 0.19 | 7.86 | 0.29 | 4.36 | 3.4 | 11.74 |
| Tryptophylarginine | 0.07 | 4.17 | 0.47 | 8.2 | -4.44 | 11.83 |
| Prolylglutamine | 0.06 | 5.19 | 0.31 | 7.02 | -2.08 | 11.85 |
| Tyrosylisoleucine | 0.17 | 6.04 | 1.19 | 7.19 | -2.16 | 11.88 |
| Leucylglycine | 0.19 | 6.55 | 0.63 | 6.16 | -0.05 | 11.89 |
| Alanylisoleucine | 0.15 | 6.1 | 1.2 | 7.47 | -2.42 | 12.22 |
| Maltotetraose | 0.23 | 5.35 | 0.31 | 7.41 | -2.14 | 12.22 |
| 3-Hydroxyhippurate | 0.01 | 6.22 | 0.12 | 6.13 | -0.02 | 12.22 |
| Prolylmethionine | 0.02 | 5.19 | 0.04 | 7.18 | -2.01 | 12.32 |
| Methionine sulfoxide | 0.33 | 6.55 | 0.61 | 6.75 | -0.47 | 12.36 |
| Tyrosylalanine | 0.35 | 6.41 | 1.01 | 7.33 | -1.57 | 12.39 |
| Alanylphenylalanine | 0.15 | 7.38 | 0.6 | 5.77 | 1.16 | 12.4 |
| Isoleucylaspartate | 0.08 | 5.67 | 0.61 | 7.47 | -2.34 | 12.45 |
| Aspartylproline | 0.2 | 7.12 | 0.46 | 6.02 | 0.84 | 12.48 |
| Valyllysine | 0.05 | 4.07 | 0.18 | 8.8 | -4.85 | 12.65 |
| Glycylleucine | 0.22 | 6.77 | 1.07 | 7.45 | -1.53 | 12.92 |
| Tyrosylglutamate | 0.17 | 6.39 | 0.62 | 7.33 | -1.38 | 12.93 |
| Aspartylleucine | 0.26 | 9.61 | 0.58 | 4.24 | 5.04 | 13.01 |
| Lysylisoleucine | 0.14 | 6.41 | 0.73 | 7.54 | -1.72 | 13.08 |
| Isoleucylalanine | 0.22 | 7.45 | 1.02 | 6.91 | -0.27 | 13.13 |
| Leu-leu-leu | 0.03 | 6.82 | 0.17 | 6.53 | 0.16 | 13.16 |
| Serine | 0.29 | 3.72 | 0.45 | 10.5 | -6.94 | 13.48 |
| 3-Methylglutarylcarnitine-C6 | 0.3 | 9.6 | 0.6 | 4.96 | 4.34 | 13.67 |
| Isoleucylmethionine | 0.04 | 10.18 | 0.56 | 4.11 | 5.54 | 13.68 |
| Lysyltyrosine | 0.16 | 7.82 | 0.38 | 6.55 | 1.04 | 13.83 |
| Phenylalanylserine | 0.12 | 6.93 | 0.51 | 7.54 | -1 | 13.84 |
| Serylphenyalanine | 0.19 | 8.09 | 0.47 | 6.42 | 1.4 | 13.85 |
| Arginylisoleucine | 0.15 | 7.38 | 0.89 | 7.53 | -0.91 | 13.87 |
| Pro-hydroxy-pro | 0.05 | 9.24 | 0.25 | 5.19 | 3.85 | 14.14 |
| Leucylarginine | 0.54 | 6.31 | 0.92 | 9.34 | -3.41 | 14.19 |
| Serylmethionine | 0.13 | 8.73 | 0.76 | 6.4 | 1.69 | 14.24 |
| Glycylphenylalanine | 0.17 | 9.13 | 0.8 | 6.28 | 2.23 | 14.45 |
| Asparagine | 0.1 | 3.95 | 0.48 | 11.25 | -7.68 | 14.61 |
| Glutamine isoleucine | 0.2 | 8.51 | 1.63 | 7.98 | -0.9 | 14.67 |
| Nacetylmethionine | 0.26 | 6.41 | 0.83 | 9.53 | -3.68 | 14.85 |
| Alanylleucine | 0.17 | 8.48 | 0.69 | 7.43 | 0.54 | 15.06 |
| prolylleucine | 0.19 | 6.59 | 0.66 | 9.47 | -3.36 | 15.2 |
| Valylvaline | 0.23 | 7.53 | 0.6 | 8.5 | -1.35 | 15.2 |
| Prolylaspartate | 0.41 | 6.28 | 0.63 | 10.24 | -4.18 | 15.47 |
| Tryptophylproline | 0.02 | 5.53 | 0.55 | 10.53 | -5.53 | 15.49 |
| Valyltryptophan | 0.06 | 8.34 | 0.29 | 7.77 | 0.35 | 15.76 |
| Threonylisoleucine | 0.14 | 7.46 | 0.77 | 9.31 | -2.47 | 15.86 |
| Phenylalanylarginine | 0.21 | 6.22 | 0.59 | 10.6 | -4.76 | 16.01 |
| 1-Methyladenosine | 0.16 | 3.87 | 0.29 | 12.63 | -8.89 | 16.05 |
| Glutathione reduced | 1.56 | 10.36 | 0.07 | 7.51 | 4.34 | 16.24 |
| Phenylalanylalanine | 0.51 | 8.12 | 1.85 | 10.55 | -3.77 | 16.31 |
| Isoleucylhistidine | 0.17 | 6.18 | 0.55 | 10.88 | -5.07 | 16.33 |
| Threonylproline | 0.05 | 7.44 | 0.3 | 9.45 | -2.27 | 16.54 |
| Glutamine-leucine | 0.12 | 10.23 | 0.43 | 6.86 | 3.05 | 16.54 |
| Arginylphenylalanine | 0.17 | 9.65 | 0.55 | 7.63 | 1.64 | 16.56 |
| Arginylmethionine | 0.31 | 9.86 | 0.68 | 7.76 | 1.73 | 16.63 |
| Isoleucylglycine | 0.12 | 8.79 | 1.26 | 9.3 | -1.65 | 16.71 |
| Arginylleucine | 0.13 | 10.32 | 0.61 | 7.27 | 2.58 | 16.84 |
| Isoleucylarginine | 0.21 | 7.42 | 0.43 | 10.39 | -3.19 | 17.17 |
| Prolylglutamate | 0.19 | 5.54 | 0.39 | 12.47 | -7.14 | 17.43 |
| Valyltyrosine | 0.1 | 8.48 | 0.48 | 9.6 | -1.5 | 17.49 |
| Threonylleucine | 0.14 | 8.22 | 0.46 | 9.94 | -2.04 | 17.56 |
| Threonylglutamate | 0.12 | 9.32 | 0.32 | 8.87 | 0.25 | 17.75 |
| Threonylphenylalanine | 0.23 | 9.41 | 0.41 | 9.05 | 0.18 | 17.82 |
| Valylphenylalanine | 0.05 | 8.41 | 0.4 | 10.04 | -1.97 | 18 |
| Prolylalanine | 0.21 | 6.25 | 0.24 | 12.3 | -6.07 | 18.1 |
| Valylleucine | 0.19 | 8.23 | 0.82 | 11.15 | -3.56 | 18.38 |
| Prolyltyrosine | 0.03 | 6.95 | 0.07 | 11.62 | -4.72 | 18.46 |
| Arginylproline | 0.1 | 9.02 | 0.3 | 10.07 | -1.25 | 18.7 |
| Serylleucine | 0.13 | 9.51 | 0.44 | 9.82 | -0.62 | 18.75 |
| Serylglutamine | 0.03 | 14.16 | 0.88 | 5.59 | 7.72 | 18.83 |
| Prolylthreonine | 0.21 | 6.16 | 0.24 | 13.32 | -7.18 | 19.03 |
| Prolyltryptophan | 0.17 | 8.19 | 0.2 | 11.3 | -3.15 | 19.12 |
| Alpha-Glutamyltryptophan | 0.19 | 12.94 | 0.85 | 7.25 | 5.02 | 19.15 |
| Isoleucylasparagine | 0.1 | 8.58 | 0.46 | 12.3 | -4.09 | 20.31 |
| Prolylphenylalanine | 0.1 | 7.31 | 0.23 | 13.67 | -6.48 | 20.65 |
| Tyrosylhistidine | 0.36 | 9.3 | 0.54 | 12.29 | -3.16 | 20.68 |
| Valylalanine | 0.22 | 8.01 | 0.57 | 13.99 | -6.33 | 21.21 |
| Threonylarginine | 0.02 | 11.84 | 0.24 | 10.61 | 1.01 | 22.18 |
| Valylglycine | 0.1 | 8.77 | 0.48 | 14.32 | -5.92 | 22.5 |
| Prolylvaline | 0.07 | 9.03 | 0.23 | 15.83 | -6.95 | 24.56 |
| Leucylasparagine | 0.31 | 10.94 | 1.2 | 15.43 | -5.38 | 24.85 |
| Valylaspartate | 0.16 | 9.78 | 0.61 | 16.86 | -7.53 | 25.87 |
| Valylglutamate | 0.11 | 10.08 | 0.56 | 16.98 | -7.35 | 26.39 |
| Tryptophylasparagine | 0.21 | 11.54 | 0.3 | 15.49 | -4.04 | 26.52 |
| Isoleucylserine | 0.16 | 12.65 | 1.2 | 15.26 | -3.65 | 26.55 |
| Tyrosylglycine | 0.11 | 13.43 | 0.47 | 13.7 | -0.63 | 26.55 |
| Phenylalanylglycine | 0.12 | 13.02 | 0.6 | 14.36 | -1.81 | 26.65 |
| Isobar-fructose-1-6-diphosphate | 0.38 | 4.49 | 0.48 | 24.52 | -20.12 | 28.16 |
| Isoleucylglutamine | 0.16 | 12.51 | 1.28 | 19.12 | -7.73 | 30.2 |
| Valylthreonine | 0.09 | 11.7 | 0.5 | 19.42 | -8.13 | 30.52 |
| Tyrosylarginine | 0.13 | 8.37 | 0.5 | 24.29 | -16.28 | 32.02 |
| Valylglutamine | 0.18 | 12.18 | 0.55 | 21.11 | -9.3 | 32.55 |
| Hippurate | 0.03 | 22.55 | 0.11 | 11.99 | 10.48 | 34.4 |
| Phenylalanylhistidine | 0.14 | 12.56 | 0.59 | 23.13 | -11.03 | 34.96 |
| Valylasparagine | 0.15 | 13.42 | 0.48 | 25.09 | -12.01 | 37.88 |
| Valylarginine | 0.08 | 12.55 | 0.22 | 28.9 | -16.49 | 41.15 |
| Valylserine | 0.1 | 14.03 | 0.45 | 31.08 | -17.4 | 44.56 |
| Arginine | 0.37 | 11.48 | 0.32 | 39.71 | -28.18 | 50.5 |
| Tyrosylglutamine | 0.36 | 22.48 | 0.88 | 36.97 | -15.02 | 58.21 |
| Mannitol | 0.44 | 16.17 | 0.96 | 58.1 | -42.45 | 72.87 |

Compounds identified as significantly different in the vaginal bacterial community state type (CST) when adjusted for smoking status. Quantile regression was conducted on centered and scaled metabolite concentrations. Significance testing was conducted with Wilcoxon rank sum test and corrected for multiple comparisons. CST-I (*L. crispatus*-dominated) and CST-III (*L. iners*-dominated) were combined for testing against CST-IV (low-*Lactobacillus*) to increase power. Metabolites that differed significantly between combined *Lactobacillus*-dominated CSTs and low-*Lactobacillus* CST-IV are displayed where q-value = <0.05 when adjusted for smoking status. Mean value of metabolite concentrations are shown. Fold change (FC) negative values indicate the decrease in non-smokers and *Lactobacillus*-dominated CSTs when compared with smokers and CST-IV, respectively.

**Table S4. Correlation between vaginal pH and detected biogenic amines**

|  | Correlation | p-value |
| --- | --- | --- |
| Agmatine | 0.17 | 0.56 |
| Cadaverine | 0.33 | 0.51 |
| Putrescine | 0.26 | 0.30 |
| Tryptamine | 0.39 | 0.46 |
| Tyramine | 0.12 | 0.48 |

Kendall’s correlation between vaginal pH and detected amines.

**Figures**


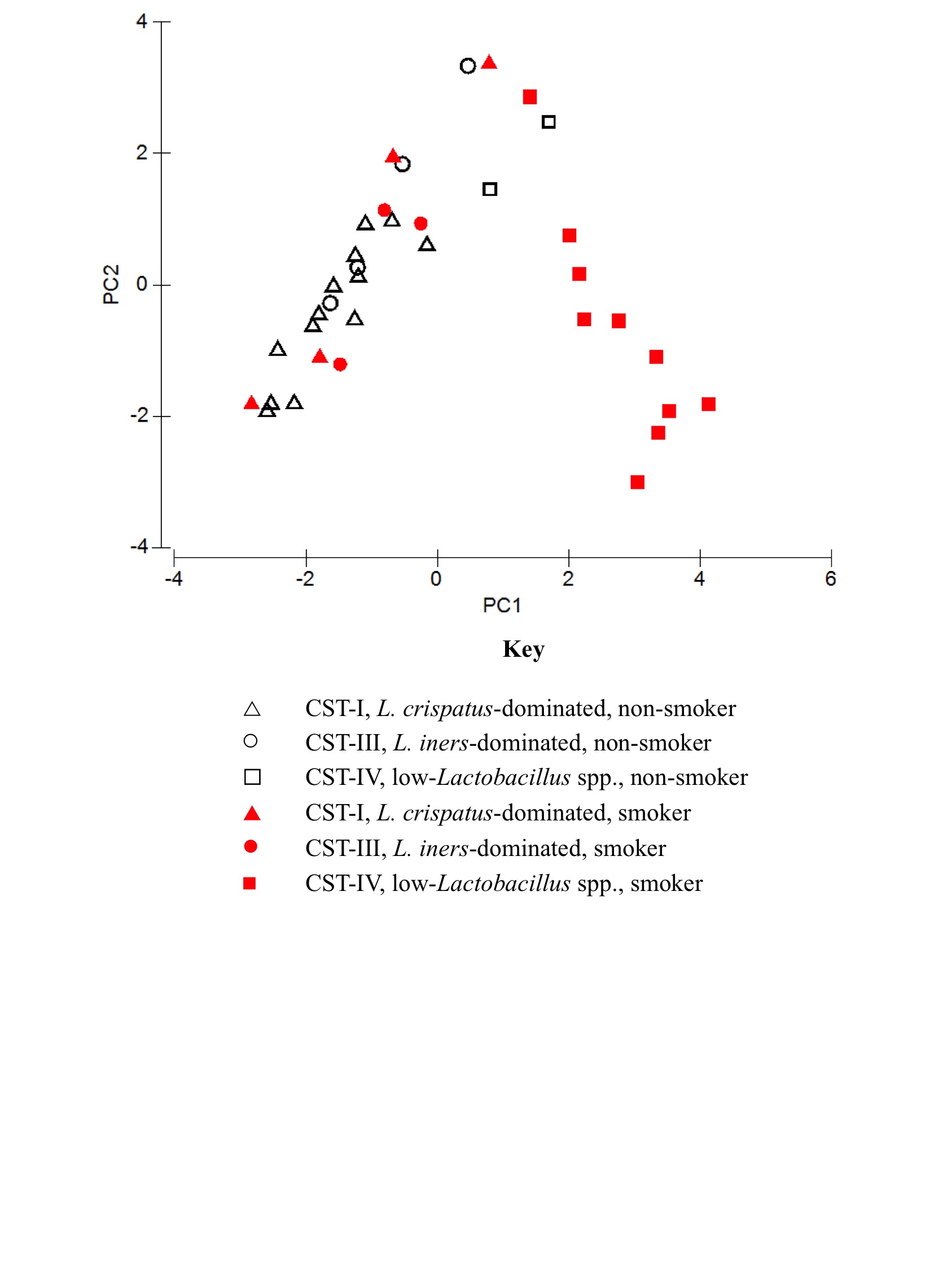


**Figure S1. Metabolome in the vagina of women grouped by community state type and smoking status**

Principal components analysis (PCA) of metabolomic data from the vagina of women grouped by community state type (CST) and smoking status. Samples are labeled as follows: individuals designated as CST-I (triangles); CST-III (circles); and CST-IV (squares) with non-smoker within each CST represented by an open shape and smoker with a shaded/closed symbol.


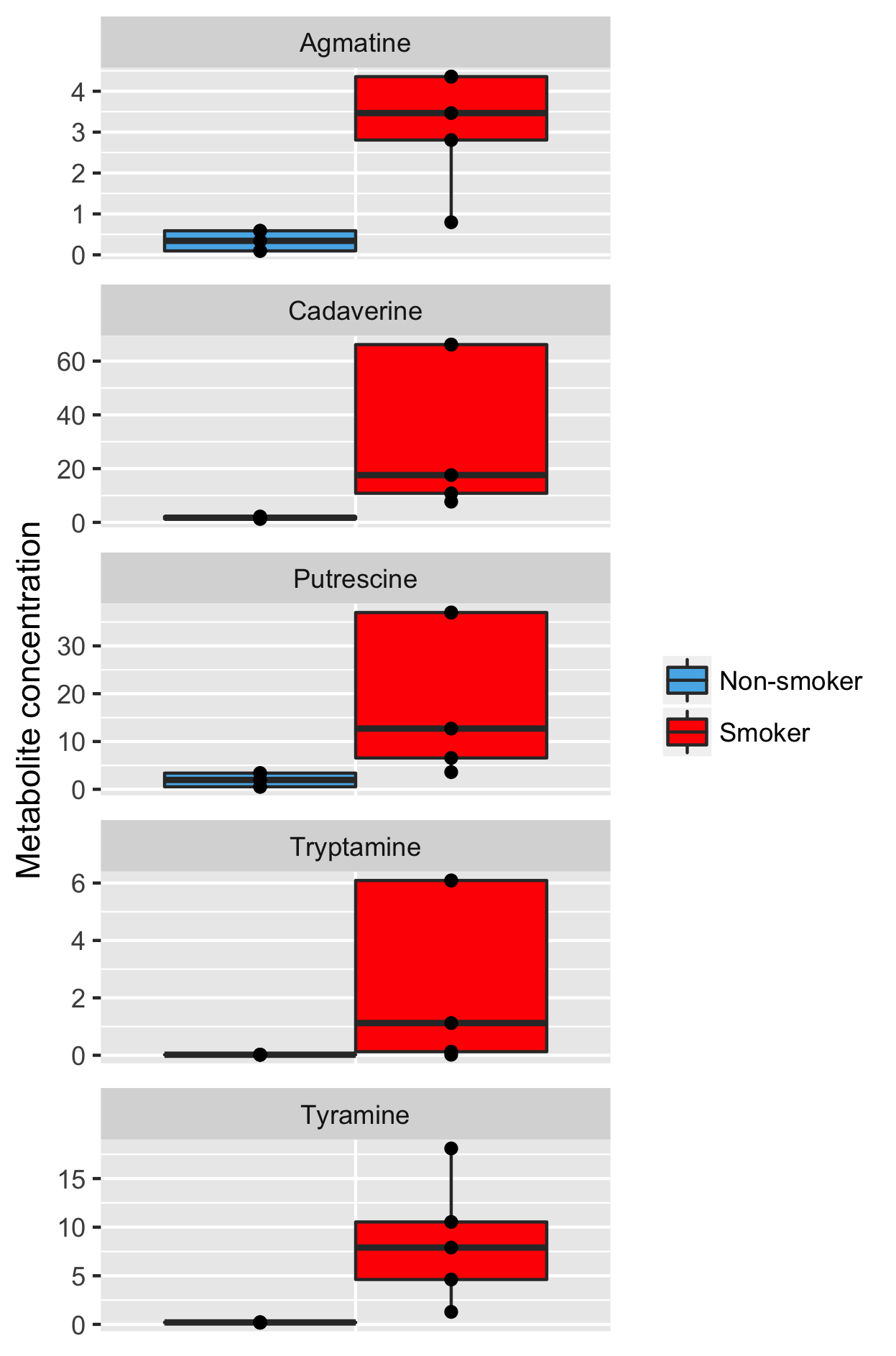


**Figure S2. Vaginal amines that differ between smokers and non-smokers in low-*Lactobacillus* CST-IV**

Boxplots display biogenic amines identified as significantly (q-value=<0.05) different in the vagina of smokers and non-smokers in women with a bacterial community state type (CST) IV low-*Lactobacillus* spp*.* Quantile regression was conducted on centered and scaled metabolite concentrations. Significance testing was conducted with Wilcoxon rank sum test and corrected for multiple comparisons.


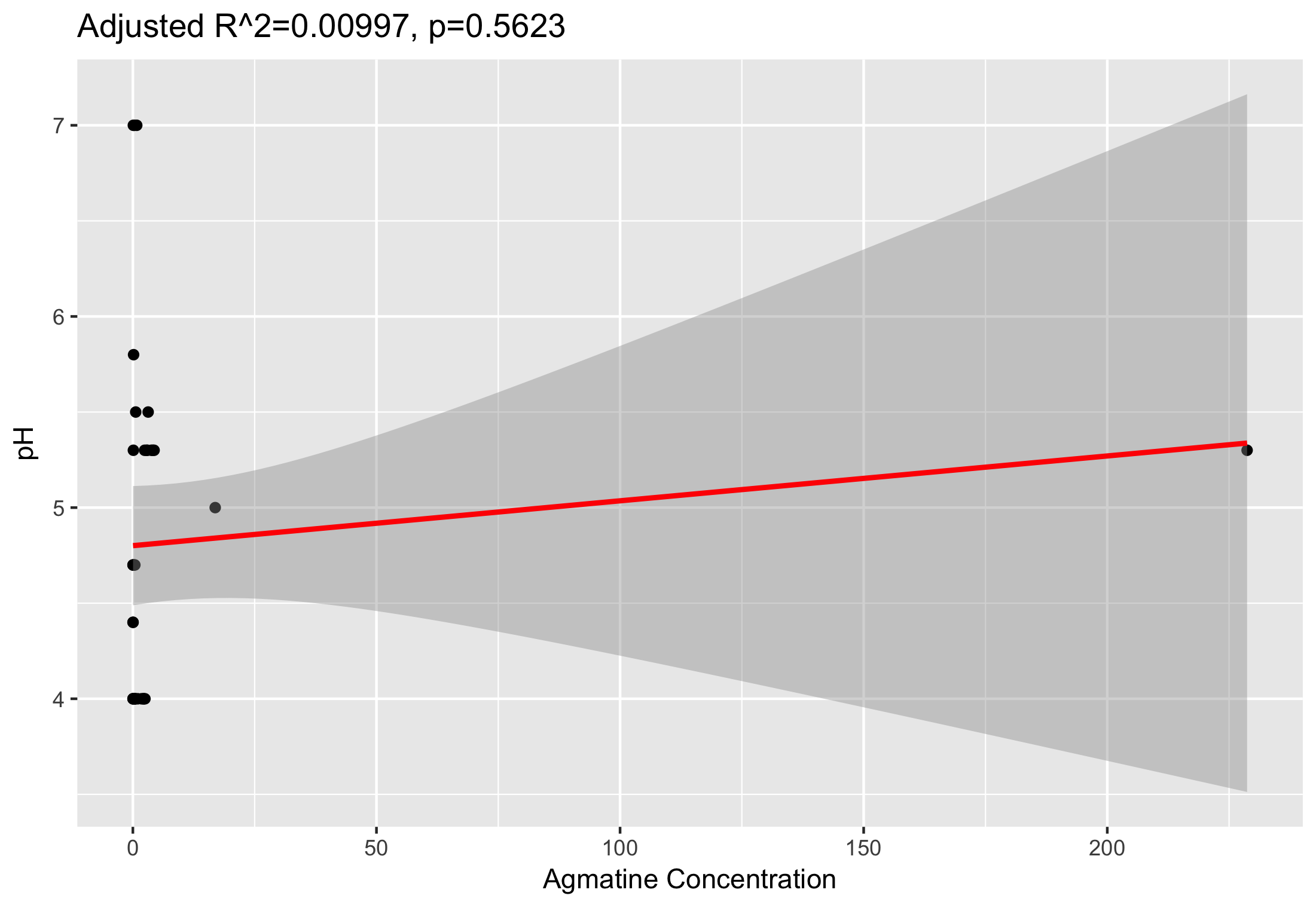


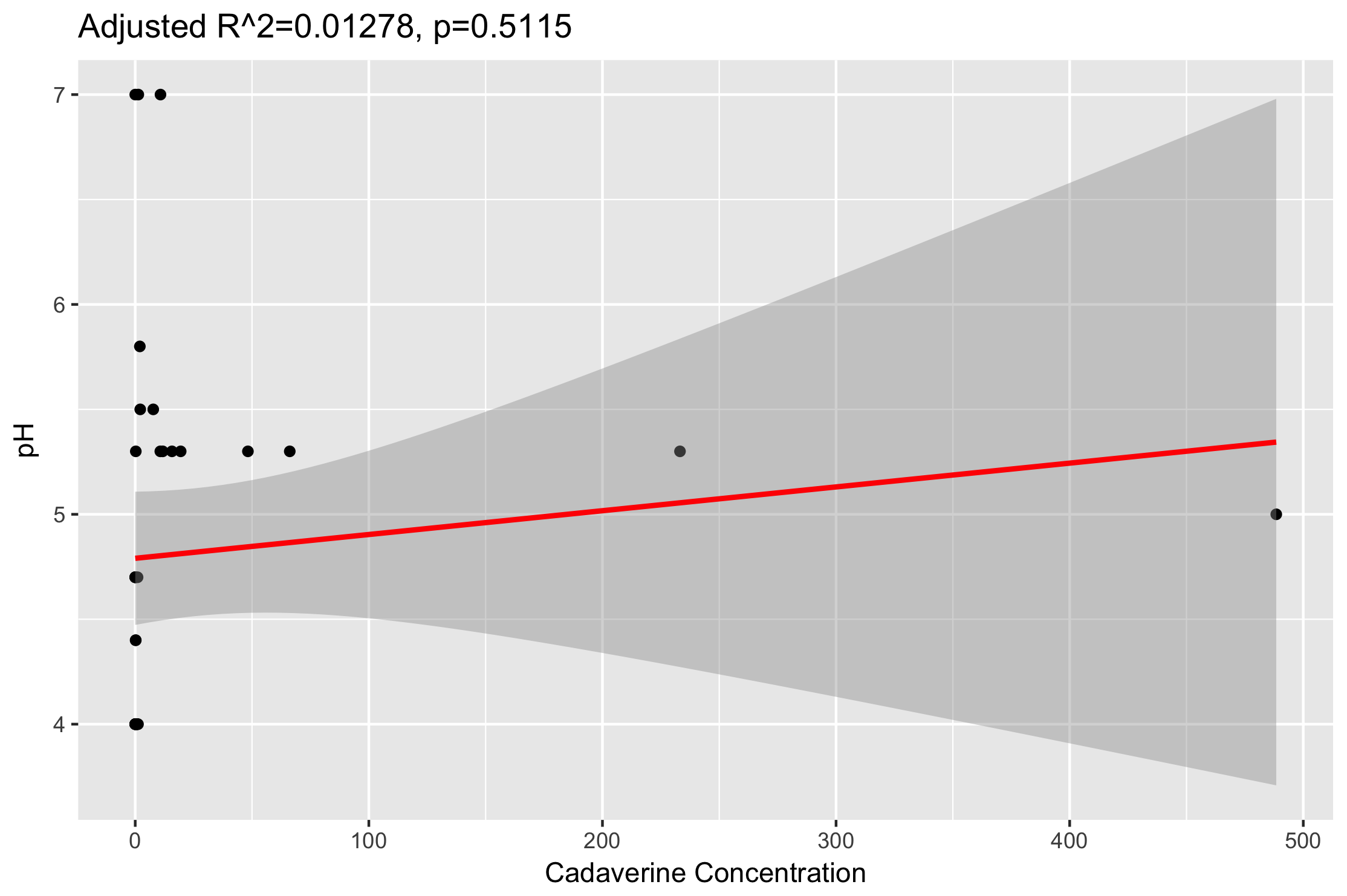


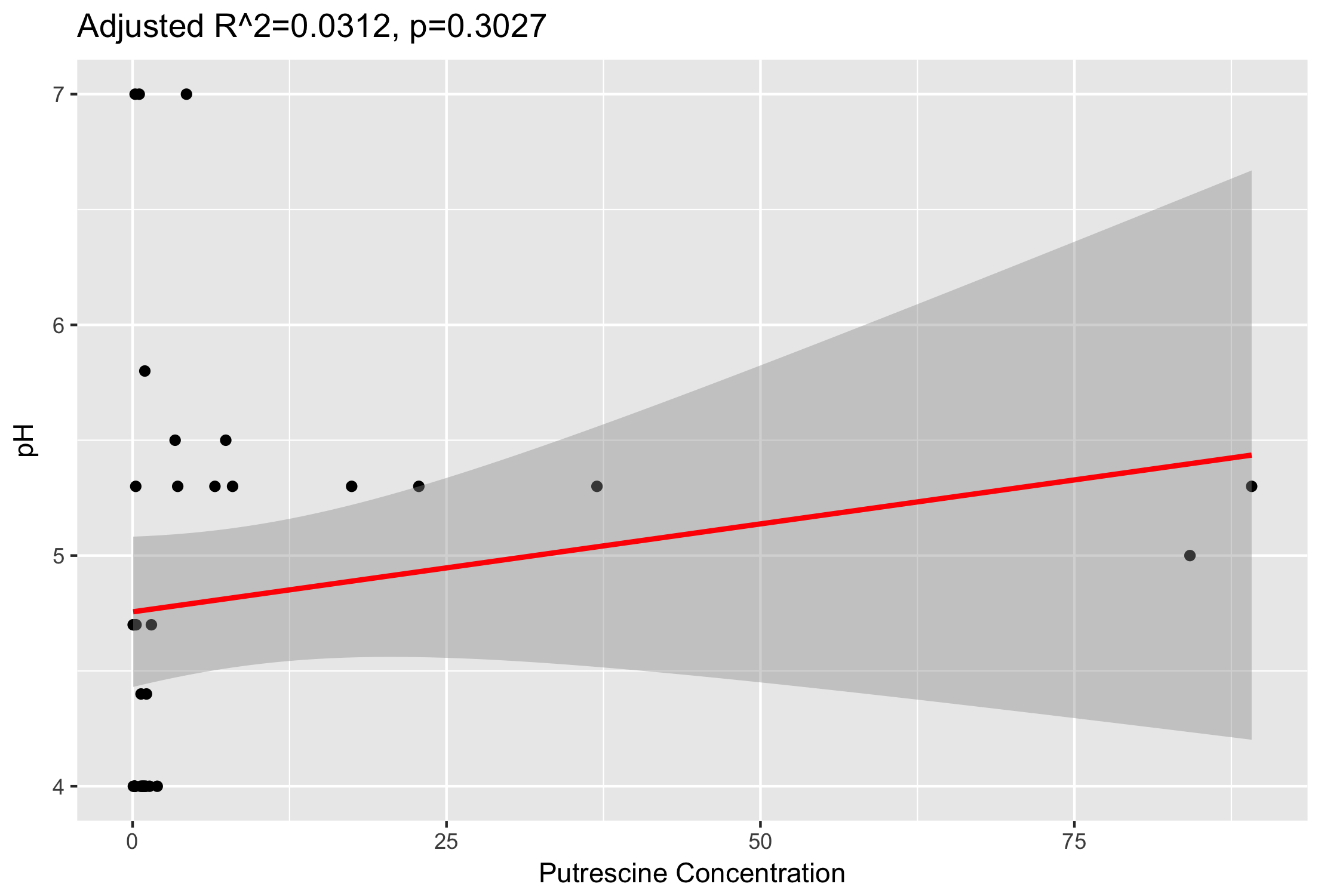


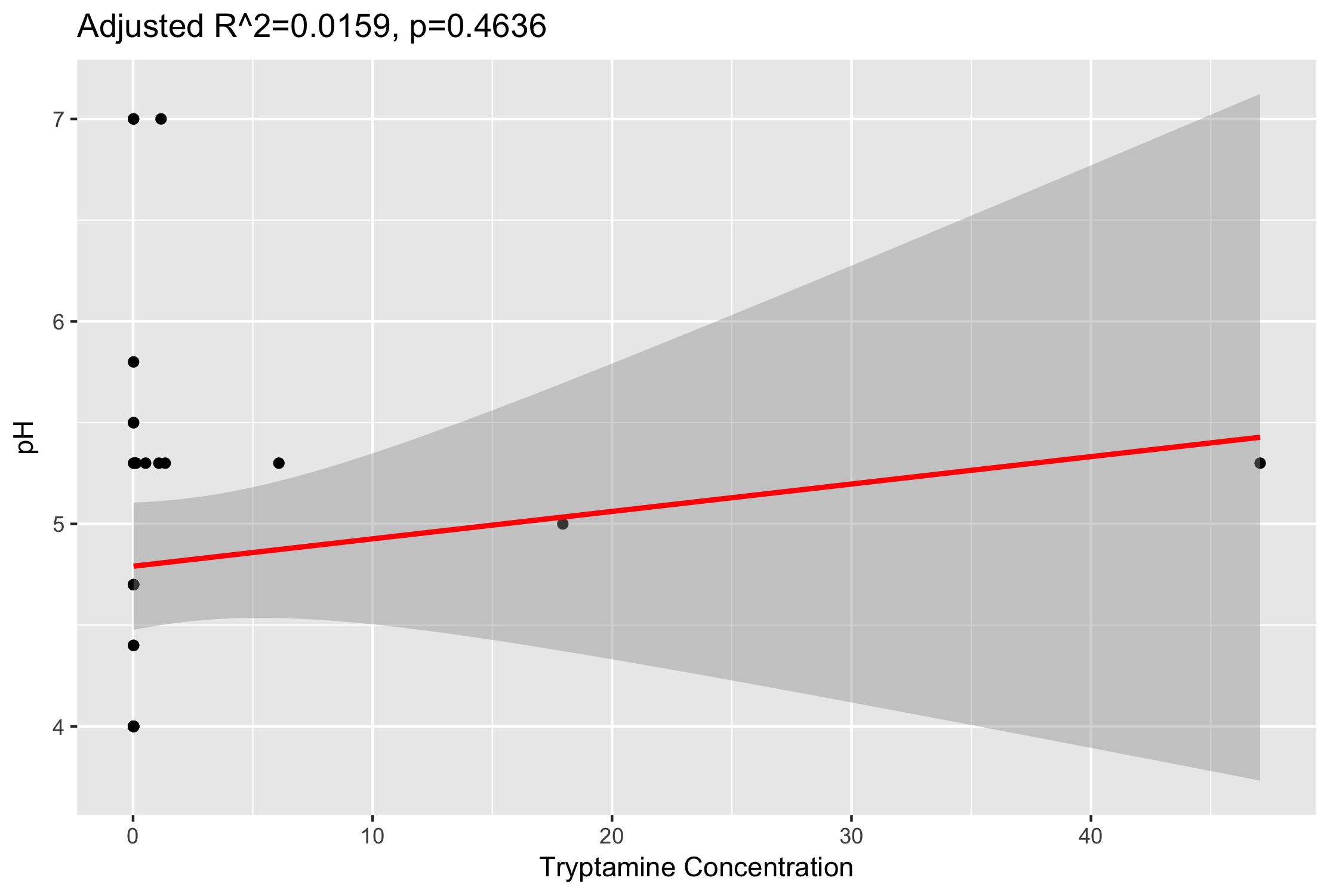


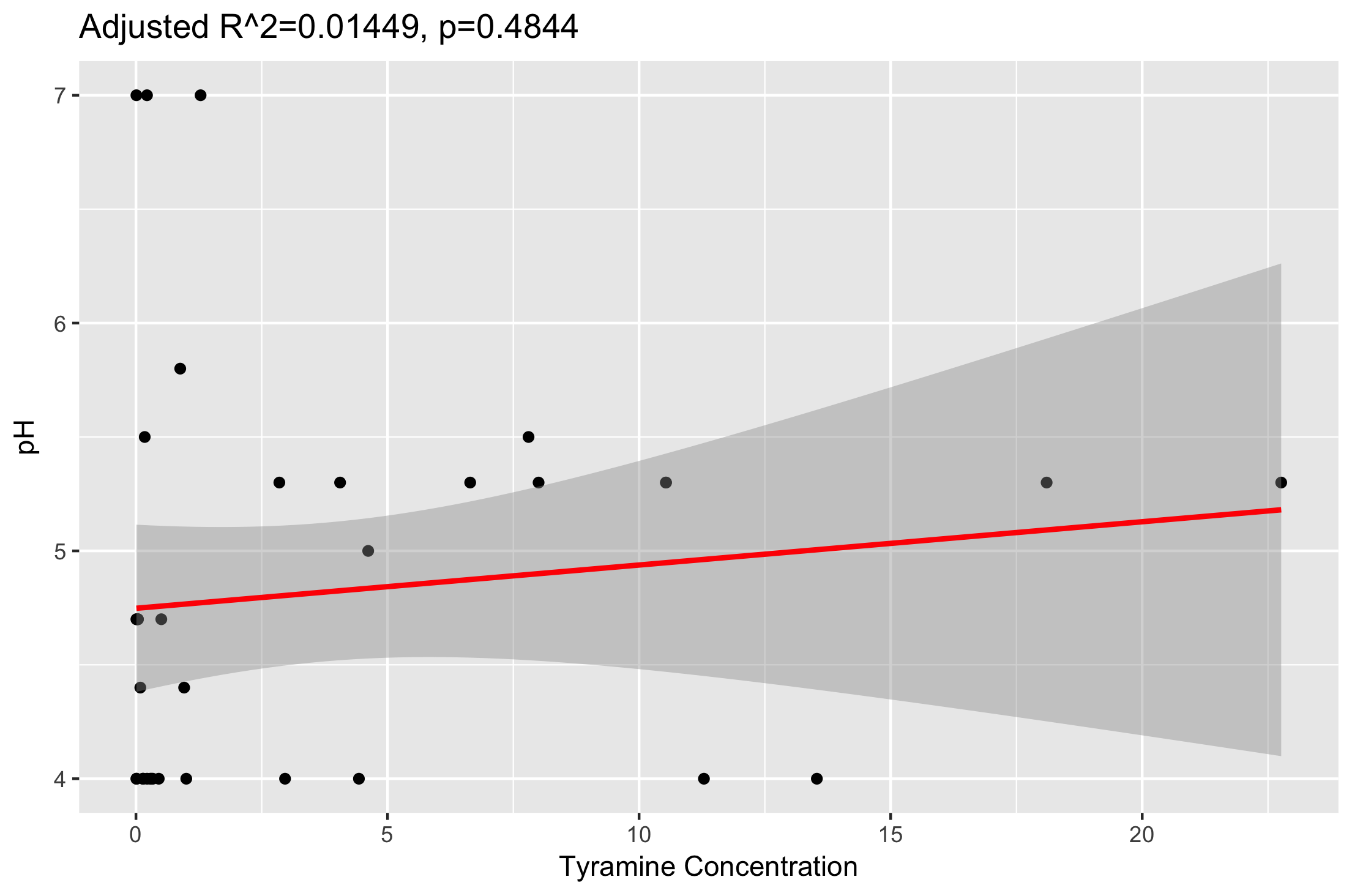


**Figure S3. Linear relationship between vaginal pH and detected biogenic amines**

Linear regression between vaginal pH and detected amines.
